# Contrast and luminance dependence of target choice and visual orientation in walking stick insects

**DOI:** 10.1038/s41598-025-90650-8

**Published:** 2025-04-10

**Authors:** Merit Meschenmoser, Volker Dürr

**Affiliations:** 1 Biological Cybernetics, Faculty of Biology, Bielefeld University, Universitätsstraße 25, 33615 Bielefeld, Germany; 2 Center for Cognitive Interaction Technology, Bielefeld University, Bielefeld, Germany

**Keywords:** Heading, Visual guidance, Target choice, Locomotion, Insect behaviour, Carausius, Navigation, Animal behaviour

## Abstract

When presented with static images, animals show robust preferences for particular visual features, and reliably turn towards and approach selected visual landmarks. In target choice paradigms, stick insects tend to approach edges with high image contrast, but also show robust orientation based on luminance alone. To better understand which stimulus features actually govern turning towards static visual targets, this study tests the relative importance of two elementary cues of spatial vision – luminance and contrast. We do so in a large open-field arena, using luminance-modulated, static 360° patterns with and without high-contrast edges. We show that target choice strongly depends on image contrast, though with a bias towards areas of low luminance. Comparison of heading directions during approach with terminal locations at the arena wall suggests an early, coarse orientation based on luminance, with subsequent steering towards high-contrast regions. When walking towards a target with high-contrast edges, the likelihood to turn away towards a Gaussian distractor image increases with decreasing edge contrast of the original target. Subjective equality of the two images occurs for an approximate 2:1 weighing of contrast and luminance, indicating that a stronger contrast-dependent edge-orientation mechanism acts in parallel with a weaker luminance-dependent phototaxis mechanism. Given the significance of stick insects as study organisms for the control of legged locomotion, future research may now test whether the two visual orientation mechanisms lead to distinct turning responses at the level of step pattern or leg movement variables.

**Supplementary Information:**

The online version contains supplementary material available at 10.1038/s41598-025-90650-8.

## Introduction

All animals with spatial vision can control the direction of locomotion relative to stationary visual landmarks or patterns. Often this includes the choice of a visual target or feature^1,2^ and its subsequent approach^3,4^. On the sensory side, this behaviour requires the detection and processing of a visual image^5^ as well as an internal rating to explain visual preferences as portrayed in orientation behaviour, e.g. in phototaxis^6,7^. On the motor side, it requires the appropriate control and coordination of locomotion^8^, particularly, as self-motion is an integral part of the feed-back loop underlying orientation^9^. Historically, sensorimotor control models^10^ have been derived from experimental data on both flying^11–13^ and walking insects^14^. Given the broad significance of visually guided approach and/or guidance relative to stationary objects in walking insects (e.g., stick insect *Extatosoma tiaratum*:^15^; ant *Formica rufa*:^16^; beetle *Tenebrio molitor*:^17^; moth *Lymantria dispar*:^18^; fly *Drosophila melanogaster*:^4,19^) it would be of general interest to understand visually induced orientation towards stationary objects in a way that links physical parameters of the visual image (e.g., contrast and luminance) to visually induced changes in intra- and inter-leg coordination.

The stick insect *Carausius morosus* is a widely used study organism in insect locomotion. It is known to adapt its motor output in response to tactile information using their antennae^20–22^ and forelegs^23^ in order to initiate climbing or turning. Given the relative complexity of a six-legged motor apparatus^24,25^ and the fact that a simple yaw turn of a walking insect may involve leg-local changes in step frequency and leg posture^26,27^, but also overall changes in inter-leg coordination^28^, it is still unclear how the range of possible turning movements are executed at the level of multiple, mechanically coupled leg joints. As a prerequisite for detailed studies on visually induced turning in legged locomotion, the present study explores the relative significance of two static image parameters - contrast and luminance - in visual target choice and orientation.

Although stick insects are nocturnal animals with fairly low spatial resolution^29,30^, they readily and reliably adapt their motor output to turn towards static visual objects^8,31^. In two-alternative choice experiments, *C. morosus* shows visual target preferences depending on size, shape and alignment within the visual field^29,32^. Qualitatively, Kalmus^33^ described edge orientation towards the boundaries of dark stripes, with a strong preference for vertical boundaries. Very similar edge orientation behaviour was found in the Australian phasmid *Extatosoma tiaratum* both when walking in a circular open field arena^15^ and in directed aerial descent^34^. However, several studies have demonstrated that the observed orientation behaviour cannot be explained based on image contrast alone but must include local estimates of luminance (“negative phototaxis” components in^17,18^ or may depend on luminance alone^15^. At a physiological level, this may relate to luminance-dependent modulation of neural encoding of image contrast^35^, calling for further experimental tests of the relative significance of image contrast and luminance on visual orientation behaviour.

The present study is a first step to complement the substantial base of knowledge on locomotion control and motor flexibility in walking stick insects^36–40^ with a systematic investigation of the relative significance of static image luminance and contrast in visual target choice and approach. We conducted a set of three experiments on freely walking *C. morosus* in a large open field arena with variable 360° images on the arena wall. While the first experiment systematically tests target choice frequencies depending on target size and contrast, the second experiment relates the walked path to the target features approached. Finally, the third experiment titrates the edge contrast that is equally attractive to a luminance-modulated target without edges.

## Materials and methods

### Animals and general experimental procedure

Behavioural experiments were carried out on adult female stick insects of the species *Carausius morosus* (Sinéty, 1901), taken from a parthenogenetic laboratory culture at Bielefeld University.

Experiments were designed to investigate the visual orientation behaviour of unrestrained walking stick insects. To this end we used a circular open field arena of 1.2 m diameter, surrounded by a vertical wall of 20 cm height. The arena size was chosen such that a stick insect of the species *Carausius morosus* would take 12 to 15 s to walk straight from the centre to the wall, an interval short enough to be repeated many times, but long enough to execute more than 10 step cycles in a steady state. Assuming typical walking speeds of 40 to 50 mm/s at 1.1 to 1.3 step cycles per second^40^, *C. morosus* was expected to execute 13 to 20 step cycles per trial. Preliminary experiments showed reliable target choice for black bars of 10° width and a preference for targets near the midline of the visual field (Supplemantary Material S1). Most specimens would readily complete 20 trials in one sequence, and several would complete up to 40 trials in a single experimental session.

Circular visual images were projected onto a translucent screen (Gerriets Transmission - creme; Gerriets, Umkirch, Germany) from the outside. For this, eight LED projectors (experiment 1: Acer K11; Acer, Hsichih, Taiwan; experiments 2 & 3: Optoma ML750e; Optoma Deutschland, Mönchengladbach, Germany) were placed below the arena and their images were projected onto the arena wall via an octagon of surface-coated mirrors. The circular projection was then divided into a sequence of eight screens, one per LED projector. The sequence of eight images were generated in Matlab (The Mathworks, Natick, USA) on a computer equipped with four graphics cards. In experiments 1 and 2 the projection screen consisted of two halves, leaving tiny gaps between them (ca. 1 mm). Although the width of these gaps subtended a visual angle of about 0.1° as seen from the arena centre (approx. 1/50 of the interommatidial angle), and none of the experiments suggested that animals reacted to their presence, a fused 360° screen of the same material was installed for experiment 3.

Animals were kept in the dark experimental room before their trial session. For each trial, they were placed in the centre of the arena such that their body long axis was pointing into one of the four cardinal directions 0°, 90°, 180° or -90°, which we will refer to as *start angle*. Typically, about forty trials were collected per animal, with pseudo-random variation of parameters as described in the specific sections for each experiment. For manually evaluated experiments, trials were scored according to the sector in which the animal reached the wall. To determine this *choice angle*, the circular wall was divided into 36 sectors of 10° width. In experiments with video analysis, the exact *choice angle* was defined by the head position as the animal first contacted the wall.

### Marker-less tracking

The motion capture system was a custom-made camera gantry (Item International, Solingen) equipped with an infrared-sensitive digital video camera (Basler A602f-2 or A602fc-2) and a manual zoom lens (Pentax). The camera could be moved along two axes, allowing the operator to follow the animal across the arena. The camera position was recorded by two contact-free linear position sensors on both gantry axes (PMS-1-A-1000-K-2410, Megatron, Putzbrunn-Munich). For further details, see^41^. Videos were recorded at a frame rate of 50 fps. Illumination came from infrared LED flashlights which were synchronised with the camera shutter via a custom-built flash trigger box (Michael Dübbert, University of Cologne). The videos were captured via fire wire (IEEE 1394) connection via the image acquisition toolbox of Matlab and stored on a PC. The camera position on the gantry, the time stamp of each video frame and an exposure signal were recorded via an AD-converter box (Data Translation DT9802) via the data acquisition toolbox of Matlab. Eight body features per video frame were tracked with DeepLabCut^42–44^. Since the goal of this study was to reconstruct (i) body position, (ii) body axis orientation, (iii) viewing direction and (iv) front leg stepping, the body features tracked were the (i, ii) mid points of the prothorax and metathorax between the front legs and hind legs, respectively, (iii) the neck and the tip of the head, and (iv) the thorax-coxa and tibia-tarsus joints of both front legs. After manual training with a set of randomly selected video frames, DeepLabCut yielded tables with 2D pixel coordinates and a confidence rating per tracked feature. These tables were then imported into Matlab for further processing. Only feature positions with a confidence rating of at least 0.95 were used (for details on confidence ratings in marker-less tracking, see^42–44^.

### Luminance and contrast measures

Luminance, L, was measured in cd/m^2^ within image patches subtending a visual angle of 1°. To do so, a Minolta Luminance Meter 1° was placed in the centre of the arena and focused on the arena wall. For bar patterns with discrete jumps of luminance, L, was measured in the centre of the bar (width 10°) and at the centre and margin of the seven background screens. For patterns with continuous modulation of luminance, L was measured in 360 one-degree steps along the arena wall.

Throughout the manuscript, contrast is generally given as Michelson Contrast of C_M_ = (L_max_ - L_min_)/(L_max_ + L_min_), as it is a common measure for local luminance modulation with discrete or continuous changes. For a given state of visual adaptation, C_M_ is a normalised measure of the spatial change in luminance. Since most of the visual patterns in this study were narrow, dark bars on a bright background, the light background dominated the adaptive state of the eye. To account for this, we give Weber Contrast, C_W_, to indicate the saliency of a local (foreground) pattern, L_FG_, on a bright background, L_BG_. Weber contrast was calculated as C_W_ = |L_BG_ – L_FG_|/L_ADAPT_, where L_ADAPT_ is the mean luminance of the arena wall. C_W_ is commonly used to describe the perceived luminance, or contrast luminance, of a stimulus patch in psychophysics^45^, but also to quantify temporal changes of luminance from an adapting stimulus to a test stimulus^35^. Since C_M_ and C_W_ differ only in their denominator, both of them are equally suited to describe changes in luminance. As the animals in our experiments experienced little or no changes in mean luminance throughout sessions, there was no reason to account for the adaptive state by using C_W_. On the other hand, C_M_ allowed for consistent normalisation of spatial luminance modulation across experiments. Although we report both contrast measures throughout, all figures and conclusions will refer to C_M_, unless stated otherwise.

### Experiment 1: contrast and luminance of plain landmarks

**Fig. 1:**
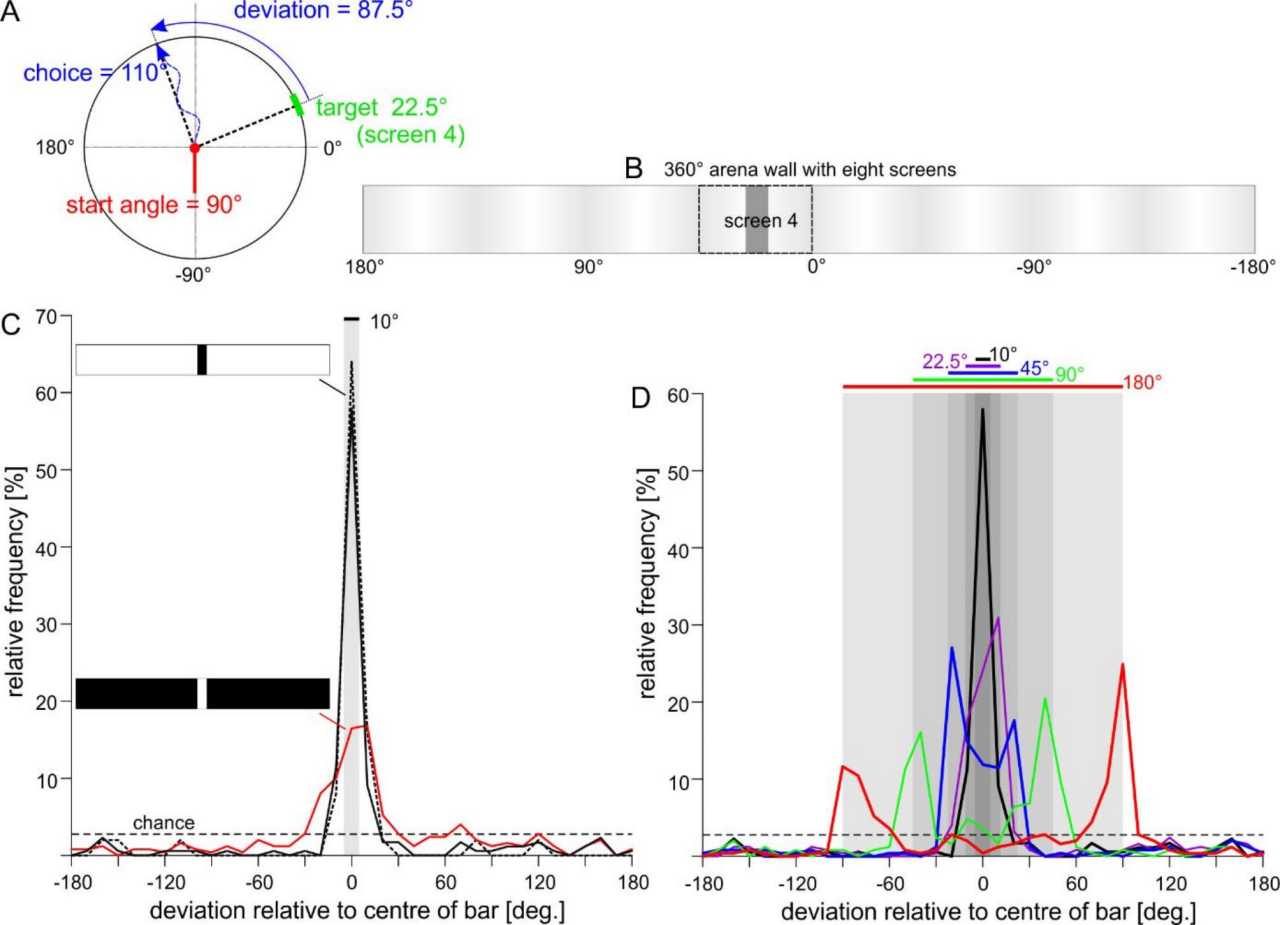
Stick insects orient towards edges. (**A**) Schematic of experiment 1 with a single landmark centred on the *target angle* (green). The *start angle* describes the heading of the animal at trial onset (red). The *choice angle* describes the head position at time of arrival at the arena wall (blue). The *deviation* is the difference *choice angle - target angle*. (**B**) The circular projection was divided into a sequence of eight screens, resulting in a periodic, low-contrast background pattern with a period of 45°. The target was always centred on one of the eight screens (e.g., on screen 4). (**C**) Part A of experiment 1: Choice frequencies for two patterns with identical Michelson contrast (C_M_ = 99%) reveal that animals preferably approached the bar, but with much lower frequency for the white bar on a black background (red line) compared to the black bar on white background (black lines). Dotted line shows result for control trials of the cohort tested on the white bar. Grey shading marks the width of the bar. Dashed line indicates chance level (1/36). (**D**) Part B of experiment 1: When presented with a black bar of varying width, stick insects show highest choice frequencies in sectors at or close to the edge of the bar. Grey shading indicates the edge locations of the five bar widths tested. Coloured lines and numbers indicate bar width.

The effect of high-contrast boundaries was tested in three parts. In part A, we used a vertical black bar of 10° width on a white background, or a white bar of 10° width on a black background. In part B, we tested black bars of variable width on white background. In part C, we rated the relative significance of visual contrast and luminance, using dark vertical bars of 10° width on a background of varying luminance. In all three parts, the bar was presented at one of eight positions, corresponding to the centres of one of the eight projection screens that assembled the 360° image on the arena wall. The centre of the bar was therefore randomly placed between angles 22.5° and 337.5° in steps of 45°. The projection of a planar image onto the curved arena wall involved slight differences in projection distance and angles of attack on the translucent screen, resulting in higher luminance at the screen centres (same angles as bar positions above) than at their margins. Therefore, although each projector image had a homogenous background, the luminance of the 360° image was modulated periodically with a period of 45°, and a Michelson contrast, C_M_, of 14.4 ± 1.9% between screen centre and margins (Fig. 1B). Bar luminance was either 3, 48, 191–611 cd/m^2^ (labelled as black, dark grey, light grey and white, respectively; the background, was either 3–611 cd/m^2^ for black or white screens, respectively (Table 1). The 10° bars were tested on one of four background luminance levels, such that both the mean luminance of the stimulus and the edge contrast of the bar were varied. Background luminance could be set to one of ten luminance levels with 2.7 ± 1.7, 10.1 ± 2.8, 24.7 ± 4.3, 48.5 ± 8.1, 80.9 ± 12.2, 129.9 ± 22.7, 191.1 ± 31.5, 267.1 ± 40.3, 354.8 ± 51.4, 475.6 ± 71.1, and 610.5 ± 89.1 cd/m^2^ (mean and standard deviation among screens). Separate cohorts of ten animals were tested for each one of 21 conditions. In three conditions with black bars of 10° width, the typical trial number per animal was 20; in all other conditions the typical trial number was 30 per animal (for details see Table 1). All of these 10 × 30-trials comprised 10 × 5 control trials with the standard condition of a black bar of 10° width on white background.

**Table 1:** Test conditions of experiment 1.

Part	Fig	L_FG_	L_BG_	C_M_	C_W_	width	*n*_all_	*n*_choice_	*n*_test_	*n*_control_	H_test_	H_control_
A	1 C	3	611	0.990	1.023	10	176	176	176	-	76.1%	-
A	1 C	611	3	0.990	30.570	10	300	299	249	50	34.1%	88.0%
B	1D	3	611	0.990	1.061	22.5	305	304	249	55	76.3%	74.5%
B	1D	3	611	0.990	1.136	45	300	294	244	50	57.8%	90.0%
B	1D	3	611	0.990	1.325	90	300	299	249	50	57.4%	84.0%
B	1D	3	611	0.990	1.980	180	305	305	249	56	52.2%	85.7%
C	2	3	10	0.538	0.714	10	195	195	195	-	37.9%	-
C	2	3	25	0.786	0.902	10	315	287	230	57	49.2%	77.2%
C	2	3	130	0.955	1.004	10	187	187	187	-	75.9%	-
C	2	48	81	0.256	0.412	10	301	264	207	57	10.1%	77.2%
C	2	48	130	0.461	0.642	10	313	228	173	55	39.9%	87.3%
C	2	48	267	0.695	0.839	10	310	286	227	59	63.4%	88.1%
C	2	48	611	0.854	0.946	10	298	287	242	45	75.6%	93.3%
C	2	191	267	0.166	0.287	10	296	277	227	50	5.7%	80.0%
C	2	191	355	0.300	0.468	10	305	288	234	54	10.7%	64.8%
C	2	191	476	0.427	0.609	10	296	276	228	48	22.4%	64.6%
C	2	191	611	0.524	0.701	10	300	296	246	50	32.9%	74.0%

**Fig. 2:**
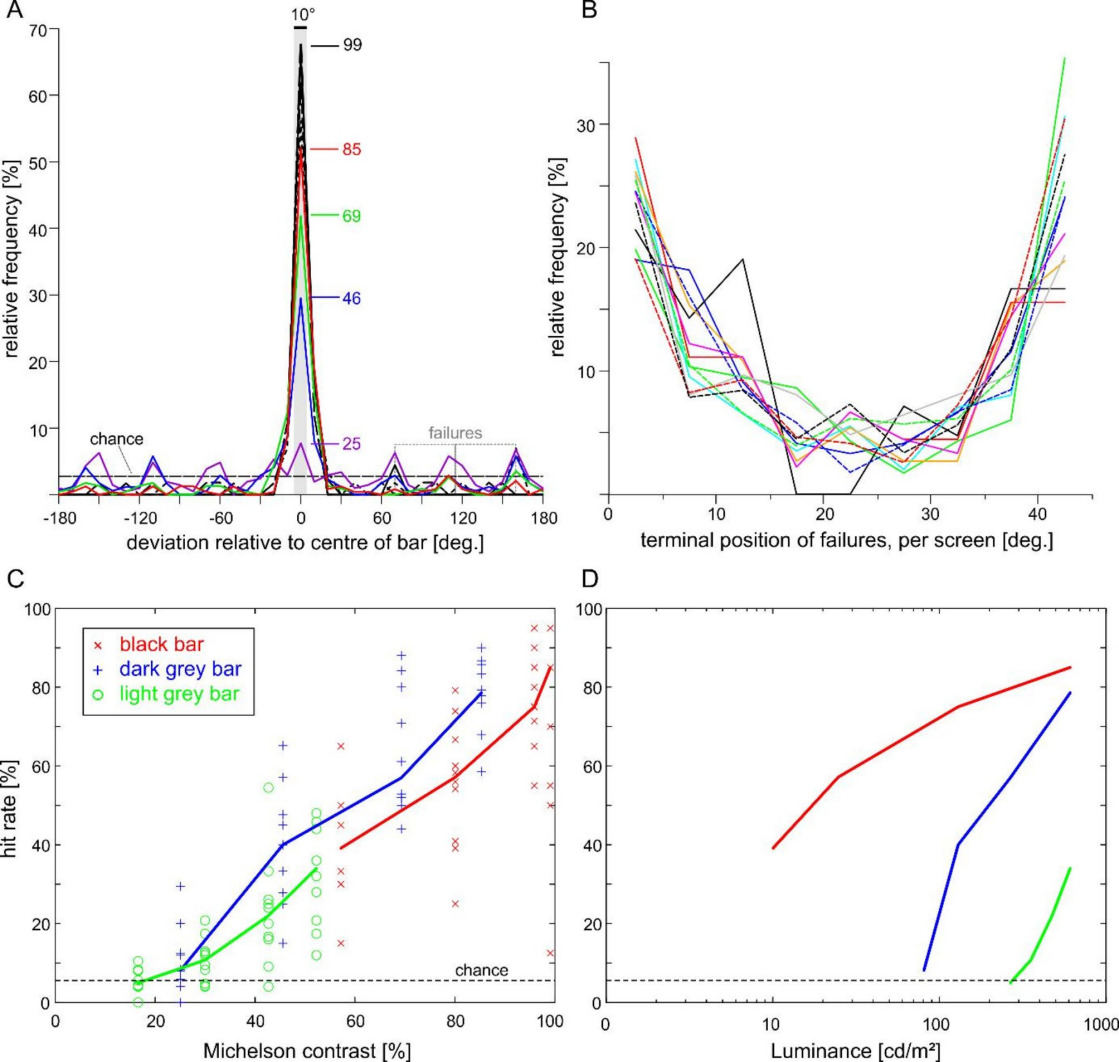
Visual orientation behaviour is governed by image contrast and modulated by luminance. Part C of experiment 1. A) Choice frequencies for a dark grey bar (48 cd/m^2^) depend on background luminance. Each colour shows the choice angle distribution of a separate cohort (*N* = 10 animals); four black lines show controls per cohort (*n* = 10 trials per cohort). Line segments with numbers indicate the Michelson contrast of the bar in front of the background). The lower the contrast, the more often animals arrived at the wall in a background sector (failure trials). Dashed horizontal line marks chance level. Dashed grey boxes indicate positions of screens 2 and 3 to the right of the bar. B) Failure trials do not terminate uniformly across background screens. Coloured lines show relative frequencies per cohort, aligned for the seven background screens per stimulus pattern. Overlay of 12 curves from independent cohorts show consistent parabolic curves with the minimum aligned with screen centres, where luminance was highest. C) Median hit rate per cohort increases nearly linearly with Michelson contrast. Chance level (dashed line) is reached when the contrast of the bar is similar to that of the periodic background modulation. Symbols show the hit rates of individual animals (*N* = 10 per column). Note that these values differ from the pooled hit rate per condition in Table 1D). Same data as on the left but plotted against the mean luminance of the background.

Test conditions of experiment 1, for parts A to C and Figs. 1C and D and 2, respectively. Test conditions were characterised by the luminance of the background (L_BG_) and the bar in the foreground (L_FG_) in cd/m^2^, as well as by the width of the bar in degrees. Michelson contrast, C_M_, and Weber contrast, C_W_, were calculated as described in the methods section. Each condition was tested with a different cohort of ten animals. Trial numbers give total number per condition (n_all_), trials with choices being made, i.e., arrival at the arena wall (n_choice_), and choices made in the test (n_test_) or control conditions (n_control_). Overall hit rates per condition are given for the test (H_test_) and control conditions (H_control_). Hits were defined as arrivals with a deviation from the bar ≤ 10 degrees.

Each animal was tested in up to ten blocks of four trials, with each block comprising a random set of the four *start angles*. *Choice frequency* was calculated as the fraction of trials that terminated in one of 36 10°-sectors. The *deviation* of an approached bar was defined as the signed difference between the *choice angle* and the *start angle*, which is equivalent to the angle of the target at trial onset (Fig. 1A). Since the *target angle*, i.e. the centre of the bar, was varied randomly in steps of 45° from 22.5° to 337.5°, whereas the *choice angle*, i.e. the head position at the wall, was rated manually in 36 sectors of 10° width, the deviation between target angle and choice angle was never zero. Accordingly, pooled *choice frequencies* have a spatial accuracy of ± 5°. With regard to particular image features, e.g., the centre of a bar, a *hit rate* was calculated as the fraction of trials in which the deviation was within the range of ± 10°. This could include two choice sectors with deviations ranging between − 2.5° and 7.5° or between − 7.5° and 2.5°. All trials with choice angles outside this “hit range“ were termed as *failure trials*.

Statistical tests for experiment 1 were calculated in R (https://cran.r-project.org/, version 4.4.1). Non-parametric two-sample tests were applied on independent choice frequencies with *N* = 10 animals per cohort in parts A and B. Dependency on bar luminance and contrast in part C was tested with a multiple linear regression of hit rate per animal against bar luminance and C_M_ or C_W_, as stated in Table 1.

### Experiment 2: luminance-modulated landmarks

To further characterize target choice as well as target approach, we used a set of six static visual patterns that differed in the number and contrast of edges. A 90° black bar with two edges (pattern *Bar*), two Gaussian luminance patterns without contrast edges (*Gauss90* and *Gauss180* with widths 90° or 180°, respectively), a ‘single-edge’ pattern (*Edge*) consisting of a sharp contrast edge next to a linear black-to-white luminance gradient (90° width) and a pattern with two edges separated by two linear luminance gradients, such that the edges differed in luminance but not in Michelson contrast (135° width, C_M_ = 20 or 50%; named *Edge20* and *Edge50*).

Four *start angles* (0°, 90°, 180°, -90°) and two *target angles*, i.e. centres of visual patterns (0° or 180°) were used in a pseudo-randomized manner. Top-view videos were collected and processed for each trial as described in section “Marker-less tracking”. A median filter (width = 5) and a Gaussian kernel (width = 5; σ = 0.83) were used to smooth feature trajectories. The *viewing direction* was determined as the intersection of the vector $\vec{\text{Neck}}, \vec{\text{Head}}$ and the arena wall. Thus, viewing direction is equivalent to the centre of the visual field. The body orientation was determined as the angle between the body axis (vector $\vec{\text{metathorax}}, \vec{\text{prothorax}}$) and the x-axis of the arena. In trials with asymmetric patterns (i.e., *Edge*, *Edge20* and *Edge50*) both mirror-symmetric versions of the pattern were used at random.

Trials in which an animal did not approach the arena wall were not analysed further. Remaining trials were categorized as ‘*at wall*’ or ‘*on target*’ trials. The target range was defined as the width of the pattern with an added tolerance of five degrees on either side. The distribution of *viewing angles* throughout whole trials were calculated as per-animal medians from *N* = 8 animals. Experiments were carried out in two cohorts. The first cohort was tested on the patterns *Bar* (two edges), *Edge* (single edge) and *Gauss90* (no edge). The second cohort was tested on the patterns *Edge20* and *Edge50* with two equal contrast edges but different mean luminance (135° width), *Gauss90* and *Gauss180* (90° and 180° width, respectively). Statistical analysis was done separately for both cohorts, using paired comparisons per animal. We collected data from 16 animals, eight animals per cohort.

To compare distributions of viewing directions during the approach with distributions of arrival positions at the wall, we tested the goodness of fit of two alternative Gaussian mixture models (GMM), one strictly unimodal and one potentially bimodal. In the unimodal variant, the Gaussians had the same mean and same standard deviation. In the bimodal variant, the two Gaussians could differ in their mean but had the same standard deviation. Both GMM were calculated using the mixtools package in R^46^. Resulting means and standard deviations are reported together with the final log-likelihood estimate of the optimisation, Akaike’s information criterion (AIC) and the χ^2^-value for goodness of fit with the empirical distribution (for both AIC and χ^2^, the lower the better) and the scaling factors lambda for the bimodal variant (the more equal, the more pronounced the bimodality). The better model was selected according to AIC, as it balances goodness of fit against model complexity. Except for the single *Edge* pattern, all other patterns contained two areas of steepest change in luminance. If edge orientation mechanisms dominated target choice behaviour, we expected to find that both distributions would be modelled best by bimodal distributions with means at or close to the ‘edges’ of the pattern. However, if phototactic mechanisms dominated target choice we expected unimodal distributions around the area of lowest luminance. For the bimodal GMM, the prior estimates for the means were set to the borders of the visual pattern and the standard deviation prior estimate was set to 20°. For the unimodal GMM, the prior estimates for the means were set to 0° and the standard deviation prior estimate was set to 20°.

### Experiment 3: two-alternative choice paradigm

To assess the relative contribution of mechanisms underlying edge orientation (contrast-dependent) or phototaxis (luminance-dependent), we designed a two-alternative choice paradigm to titrate variable edge contrast against a fixed luminance-modulated pattern without contrast edges. Animals were first cued to walk towards a grey bar (the *original target*) and then offered an additional *distractor target* that was shifted by either + 60 or -60 degrees. Two *start angles* (0°, 90°) and two distractor positions were used in a pseudo-randomized sequence. We then used an adaptive staircase method to change the grey level of the *original target* in order to obtain the edge contrast of the bar at which the Gaussian *distractor* (without edges) was no longer preferred. The *original target* was 20° wide, the Gaussian *distractor* was 60° wide with σ = 10°, i.e., with the luminance gradient being steepest at ± 10° on either side of the centre. Interspersed control trials used a grey bar of equal contrast and size as the distractor, thus testing the preference for maintaining a straight course despite the occurrence of a distractor. The initial contrast of the staircase was determined for each animal individually. For this, two criteria had to be met. First, the initial contrast had to be sufficiently high to make the animal walk towards the *original target* reliably. Second, the contrast had to be low enough for the animal to turn towards the Gaussian *distractor*. After each trial in which the animal approached the Gaussian *distractor*, the Michelson contrast of the *original target* (bar) was increased by a fixed step size (+ 3.1%). After two subsequent approaches of the *original target* (bar), the contrast of the bar was decreased by a fixed step size (-3.1%), thus following the “transformed 1-up-2-down method”^47^. To estimate the critical contrast at which both patterns were equally attractive, we coded the decisions of the resulting staircase as 0 (Gaussian) or 1 (Bar) and fitted a logistic function to all trials per animal, using maximum likelihood optimisation in Matlab. Each sigmoid function yielded two values: (1) The point of subjective equality (PSE) which indicated the contrast at which there is a 50% cance for either target. (2) Second, an adjusted PSE that accounted for the animals’ preference for the original target. The adjusted PSE was the contrast at which the *Gauss* pattern achieved the same choice rate as the distractor *Bar* during control trials. The unadjusted PSE at 50% cn be viewed as a ‘behavioural threshold’. At this contrast level the probabilities for both targets are equal although the patterns are likely not perceived as equal due to the bias for the pattern straight ahead. Individual staircases and sigmoid functions were acquired for 9 animals.

To relate the critical contrast to features of the visual images as seen by the animals, we needed to account for the spatial filter characteristic of the animals’ compound eyes. To this end, we convolved the measured luminance pattern along the arena wall with a Gaussian kernel designed to mimic an average acceptance angle of 4.7° per ommatidium^30^. Since acceptance angles are specified as full width at half maximum (FWHM) of the Airy disc and the FWHM of a Gaussian equals 2.355 × σ, we applied a kernel with σ = 2°. Maximum perceived contrast of a pattern was defined as the maximum slope, i.e. steepest luminance gradient, of measured luminance (ML) after convolution with this kernel. As a measure of mean luminance between positions x_1_ and x_2_, we computed the approximate integral of the ML for both patterns according to:

$$\:{\int\:}_{\varvec{x}1}^{\varvec{x}2}\left(1-\varvec{M}\varvec{L}\left(\varvec{x}\right)\right)\:\varvec{d}\varvec{x}\:,$$

equivalent to the area beneath the ML curve.

## Results

### Effects of contrast and luminance on target choice

Using a standard bar width of 10°, experiment 1 was to test whether visual orientation towards static landmarks was related to the contrast of the landmark’s edges or rather its luminance. In part A of this experiment, we varied the mean luminance of the image without changing the Michelson contrast, C_M_, of the edges. To do so, we inverted the image into a white bar on a black background and tested how this affected the choice frequency of the 10° bar, but also the distribution of choice angles relative to the bar. If visual orientation was related to image contrast, we would expect animals to approach the edges of the bar, resulting in high choice frequencies near the bar for both stimuli. Moreover, if C_M_ of the bar’s edge was a good predictor, we would expect choice frequencies to be equal for both stimuli. If, on the other hand, visual orientation was related to image luminance, we would expect animals to approach the dark sections of the arena wall. Accordingly, the choice angle distribution should show a sharp peak centred on 0° deviation in case of the black bar, and a much broader peak opposite to the white bar, i.e. centred on the 350° wide black section of the wall. The results in Fig. 1C show that for both contrast and luminance, only one of these expectations proved to be correct:

Regarding image contrast, choice angle distributions indeed peaked near the bar, with hit rates of 76.1% and 34.1% for black and white bars, respectively (Table 1, rows 1–2). However, the white bar was much less attractive than the black bar, such that choice frequencies at the bar were significantly higher for the black bar than for the white bar (mean difference of *N* = 10 animals per cohort: 37.6% points; Wilcoxon’s rank sum test, *p* < 0.001). This difference was not due to differences between cohorts, as the same animals that showed the low hit rate for the white bar reached a hit rate of 88% in control trials. Note that Weber contrast, C_W_, of the white bar’s edges was about 30 times that of the black bar, such that the mismatch with expectation was more pronounced for Weber contrast than for Michelson contrast (Suppl. Mat. 2).

Regarding image luminance, the choice angle distributions for the black bar clearly peaked at the centre of the bar, with a peak value of 64% at 0°, as expected. In case of the white bar, the peak value of 16.9% was recorded at 10°, nearly opposite to its expected location. As the shallower, broader peak at the white bar could have resulted from animals having a bias towards the dark side of the edge, we compared the choice frequencies at the bar against choice frequencies next to the bar. To do so, we compared choice frequency pairs per cohort. In case of the white bar, animals were significantly less likely to choose the bar than one of the adjacent black 10° sectors, by 10.5% points (Wilcoxon’s test for matched pairs, *N* = 10 animals, *p* = 0.018). In case of the black bar, choice frequencies at the bar were significantly higher at the bar, by 35.3% points (Wilcoxon’s test for matched pairs, *N* = 10, *p* = 0.006).

We conclude that stick insects tend to approach high-contrast regions within a large-field visual stimulus. The relatively narrow peak for the black bar and the broader peak found for the white bar could be related to a tendency to prefer the darker side of a contrast edge.

If this interpretation was correct, the animals’ heading direction should depend very strongly on the location of the edge, but with a bias towards lower luminance. Accordingly, we expected that the shape of the choice angle distribution should change with increasing width of the black bar in two ways: First, the distribution should become bimodal beyond a certain bar width. Second, the choice frequencies on the dark side of either contrast edge should always be larger than that on the light side. To test this, part B of experiment 1 comprised another four cohorts of ten animals exposed to visual images with black bars of different width (22.5° 45°, 90° and 180°), using one bar width per cohort. Control trials were recorded for each cohort. The results clearly confirmed both expectations (Fig. 1D; rows 3–6 in Table 1). First, the choice angle distributions were unimodal for the 22.5° bar, as for the 10° controls, but bimodal for bar width 45° or larger. Second, choice frequencies of the 10° sectors on adjacent dark and light sides of the edges were always higher on the dark side (Table 2). Note that unbiased orientation towards contrast edges should have led to four equal choice frequencies per row in Table 2. This was not the case. Instead, animals showed a consistent bias towards lower luminance. Pair-wise tests of choice frequencies per animal revealed that choice frequencies were significantly higher in the black 10° sectors than in the juxtaposed white 10° sectors in case of the 22.5, 45 and 90° bars, but not for the 180° bar (Wilcoxon’s test for matched pairs, *N* = 10 animals per cohort; 22.5°: *p* = 0.006; 45°: *p* = 0.006; 90°: *p* = 0.025 ; 180°: *p* = 0.066).

**Table 2:** Choice frequencies in 10° sectors immediately adjacent to contrast edges.

Bar width	White, left	Black, left	Black, right	White, right
22.5	4.0%	17.7%	30.9%	3.2%
45	1.2%	27.0%	17.6%	1.2%
90	11.2%	16.1%	20.5%	9.6%
180	0.4%	11.6%	24.9%	2.8%

Percentages show pooled choice frequencies of Fig. 2D (*N* = 10) for adjacent points on the black and white sides of the edge. Wilcoxon’s test for matched pairs for *N* = 10 animals per cohort was statistically significant for bar widths 22.5, 45 and 90°.

At the same time, the hit rates of the control trials per cohort ranged between 74.5% and 90.0%, i.e. lay in the same range as in part A (Fig. 1C). We conclude that stick insects favour the dark side of a contrast edge. For bars wider than 40°, the choice frequency peaks on either side of the bar. For narrower bars, the two peaks may fuse to a single, sharp peak that is centred on the target.

The preference for the edges of a black bar suggested that it was image contrast rather than luminance that governed visual orientation in freely walking stick insects. In order to measure contrast dependence directly, we systematically varied both contrast (C_M_: 17 to 99%; C_W_ = 0.29 to 1.02) and luminance (10 to 611 cd/m^2^) over a set of twelve stimuli with 10° bars. Eleven further cohorts of ten animals were tested, nine of them including control trials. Together, the twelve visual stimulus patterns tested combined one of three luminance levels for the bar with one of four luminance levels for the background (Table 1, rows 7–17). We expected that choice frequency would increase with increasing image contrast irrespective of bar luminance, but differ strongly with regard to the mean luminance of the stimulus. Furthermore, we expected that the “failure trials”, i.e. trials in which an animal did not approach the bar but some other part of the arena wall, would be evenly distributed across the sectors with background luminance.

The results clearly show that choice frequency for a bar with fixed luminance decreases with increasing background luminance and, therefore, decreasing contrast (Fig. 2A, with data for “dark grey bar”, along with controls). When plotting hit rates per animal for all twelve stimuli, we found a nearly linear dependency of median hit rate on image contrast (Fig. 2C) and very similar contrast dependency curves for the three types of bar. The same was true for Weber contrast (Suppl. Fig. S2). When the same data were plotted against background luminance (which was very close to mean luminance since the background covered 350° of the image), choice frequency was also clearly dependent on luminance, but with very different luminance dependency curves for the three types of bar (Fig. 2D). This was reflected by the results of a multiple linear regression of hit rate per animal against the independent variables bar luminance and contrast, which explained 72.9% of the total variance in case of C_M_ and 72.5% in case of C_W_. For both model variants, the effect of contrast was highly significant (*p* < 0.001), whereas the effect of luminance was non-significant (*p* > 0.285).

Regarding the failure trials, we noted that many choice angle distributions had very clear peaks in sectors with background luminance (Fig. 2A). Particularly for images with low-contrast bars, choice frequency distributions showed periodically occurring peaks that could reach twice the expected chance level (e.g., purple line in Fig. 2A for dark grey bar with C_M_ = 25%). The apparent periodicity of these local peaks led us to the assumption that there might have been a correlation with the moderate, periodic modulation of background luminance (see schematic in Fig. 1B). Accordingly, we collected all failure trials of a particular cohort and plotted the relative frequency of all choice angles over their position within each one of the eight projection screens. The resulting twelve graphs (one per cohort) took a parabolic course with the frequencies at the screen boundaries being always at least twice that in the screen centre (Fig. 2B). Other than expected, this shows that animals did not approach the background in a uniform way. Instead, stick insects appeared to respond consistently to the modulation of background luminance, preferring the slightly darker regions over the slightly lighter regions. We conclude that stick insects choose their direction of heading according to image contrast. When approaching a region without contrast edges, they show a consistent preference for darker image regions, irrespective of the mean luminance.

### Orientation towards visual objects with two, one or no contrast edges

**Fig. 3:**
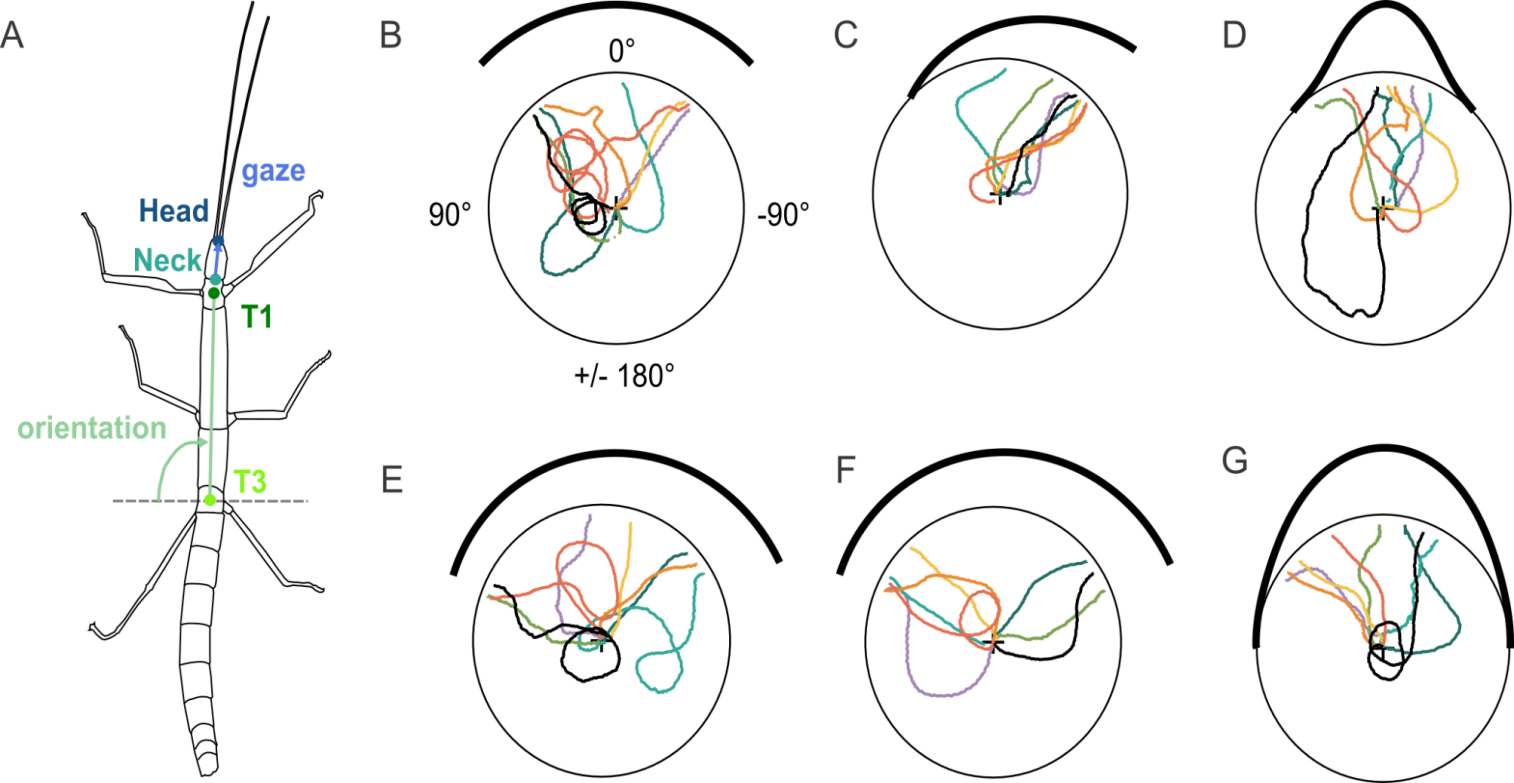
Approach trajectories for different visual stimulus patterns. (**A**) Body features tracked for further analysis. Green and blue lines show the axes used to compute body orientation and viewing direction, i.e. gaze angle, respectively. (**B-G**) Coloured lines show the prothorax (T1) trajectories of the first pattern approach of each animal (*N* = 8 per pattern, colours correspond to individual animals), with the circle representing the arena wall. Luminance of visual patterns is displayed by thick black lines such that increasing distance from the circle indicates increasing darkness (zero distance indicates “white”. Start orientation of the body axis was randomized across trials, as reflected by different initial orientation of the trajectories at the centre. (**B**) Two-edge pattern *Bar*. (**C**) Single edge pattern *Edge*. (**D**) Two edge pattern with luminance modulation and contrast edges with C_M_ = 50%; *Edge50*. (**E**) As D but with C_M_ = 20%; *Edge20*. (**F**) Gaussian pattern of width 90° (σ = 15°; *Gauss90*). (**G**) Gaussian pattern of width 180° (σ = 30°; *Gauss180*).

Given that contrast has a dominant role in visual orientation in stick insects, though with a bias towards low luminance, we wondered whether the point of arrival at the arena wall was set early or late during the approach phase. In the context of feedback-controlled taxis behaviours (luminance-dependent negative phototaxis or contrast-dependent photohorotaxis) this is related to the question, whether the target choice that determines subsequent feedback-controlled approach behaviour occurs at the start of a trial (and only once) or may occur later (and, potentially, multiple times), e.g., after an initial exploratory phase. To address this question, we recorded the entire approach behaviour towards landmarks with different patterns of luminance modulation and different numbers of contrast borders. To relate the final choice of a target to the walking path during approach, we motion-captured freely walking animals using overhead video recordings and marker-less tracking. The obtained 2D-position data (Fig. 3A) allowed us to describe the approach behaviour by means of time courses of head position as well as head orientation and, therefore, the viewing direction, i.e., centre of visual field. As our aim was to examine the relative importance of negative phototaxis (i.e., luminance-dependent) and edge orientation (i.e., contrast dependent) mechanisms, we designed a set of six luminance patterns comprising either two (e.g., *Bar*; Fig. 3B), one (*Edge*; Fig. 3C) or no high-contrast edges (*Gauss*; e.g. Figure 3D). Patterns with two contrast edges additionally varied in luminance while maintaining the same edge contrast (*Edge50*, *Edge20*; Fig. 3E, F). Patterns without edges also varied in width (*Gauss90*, *Gauss180*; Fig. 3D, G).

Since Zeng et al.^15^ showed that 1st instar nymphs of the Australian phasmid *Extatosoma tiaratum* can make decisions based on luminance alone, we included two Gaussian luminance patterns that lacked any high-contrast edges but comprised two regions with continuous luminance gradients. Owing to the different widths, the luminance gradients were steeper in the narrow (90°) than in the broad (180°) pattern. Assuming that visual contrast, i.e. spatial change in luminance, was the main determinant of visual orientation behaviour, we expected that animals would approach the regions where the luminance gradient was strongest, resulting in a bimodal distribution of arrival positions at the arena wall. Similarly, we expected bimodal distributions for the three patterns with two equal-contrast edges (e.g., the *Bar* pattern), with a preference for the edge with lower mean luminance in *Edge50* and *Edge20* patterns. In the *Edge* pattern, where the locations of maximum contrast and minimal luminance coincide, we expected a unimodal distribution of arrival positions at the wall. Assuming that the animals decided early on in a trial, which part of the pattern to approach, we expected very similar distributions for both the viewing direction per trial and the final position at the arena wall. Assuming that low-contrast patterns were less attractive than high-contrast patterns, we expected lower probability to be on target, reduced speed and increased path curvature (i.e. less straight paths) for patterns without edges (*Gauss90*,* Gauss180*) or with low contrast edges (*Edge50*,* Edge20*) due to the reduced cue strength.

**Fig. 4:**
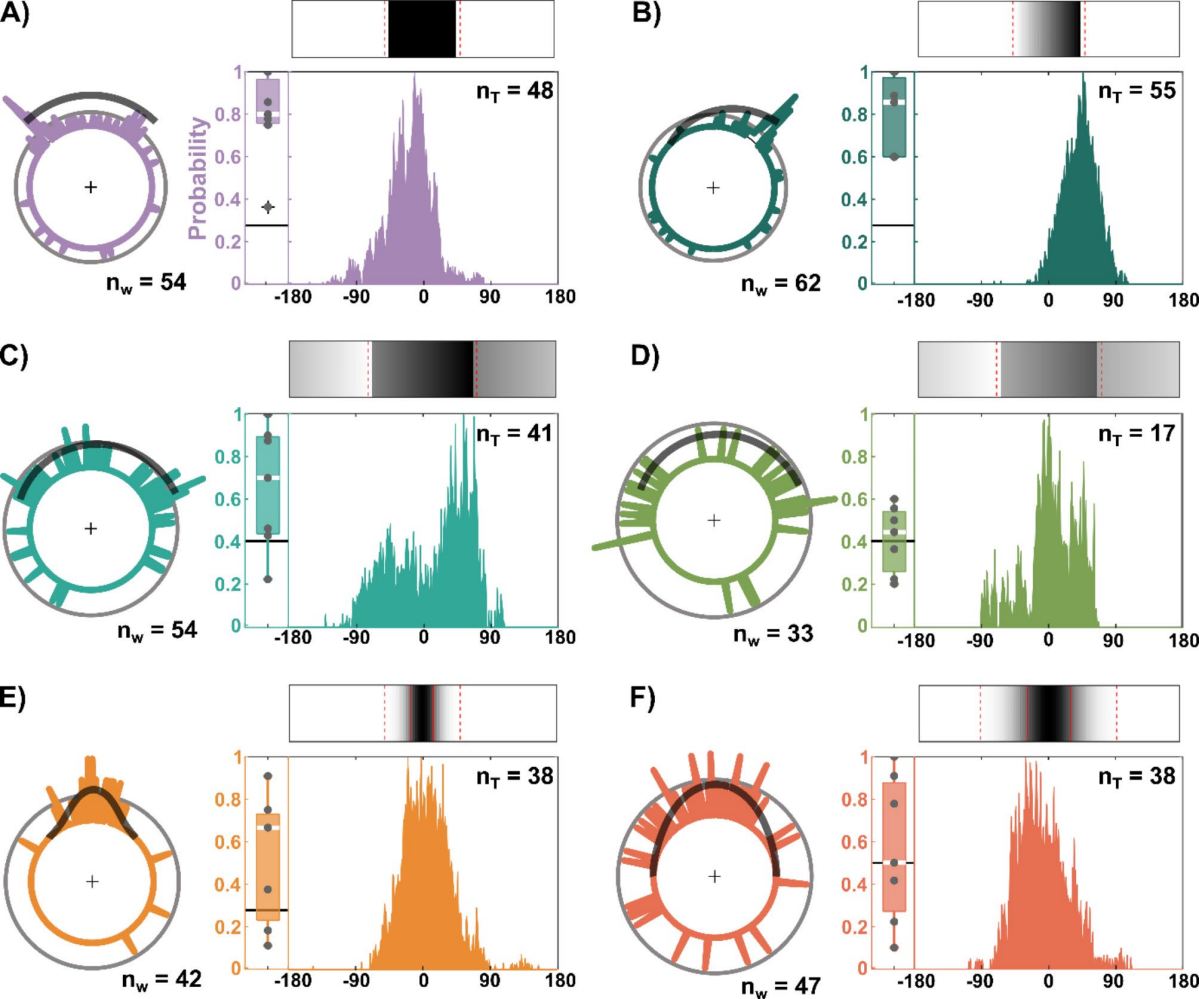
Low luminance predicts viewing direction while contrast predicts where animals reach the wall. Each panel comprises four summary graphs for a particular visual pattern: (1) Circular distributions show the final head positions of all trials that terminated at the arena wall. The number of these “at wall“-trials is given below (n_w_). Visual patterns are displayed as thick black lines, with luminance being proportional to the distance from the inner circle. Thin grey circles show the 95% confidence level of a random distribution. (2) Box plots show the likelihood per animal to be on target, given the trial terminated at the arena wall (grey circles show per-animal means). The target range was defined as the width of the pattern with an added tolerance of five degrees to either side (marked as dotted red lines within the visual pattern). White band indicates median, the edges of the box are the 25th and 75th percentiles. Outliers are indicated by an underlying ‘+’. Black lines indicate chance level. The chance level was adjusted to account for the difference in target width (target width, including a total of 10° tolerance). (3) Rectangular histograms show the median probability distributions of viewing direction per animal (N = 8) for *on-target* trials (n_T_) only (see Suppl. Mat. S3 A for probability distributions of viewing direction for all *at-wall* trials). Probability distributions were normalized by the maximum probability to facilitate comparison. (**A**) *Bar* pattern with two high-contrast edges. (**B**) Pattern *Edge* with single high-contrast edge. (**C**) Pattern *Edge50* with two edges of same contrast (50%) but different mean luminance. (**D**) As C but with 20% contrast; *Edge20*. (**E**) Narrow Gaussian pattern (90° width). Red lines highlight steepest change in luminance. (**F**) Wide Gaussian pattern (180° width). Red lines highlight steepest change in luminance. For a quantitative comparison of unimodal and bimodal Gaussian mixture models for distributions of viewing direction and position at wall, see Suppl. Mat. S3.

Individual trajectories varied between animals (Fig. 3, B-G) but also across trials. Trajectories often revealed multiple turns, and sometimes comprised complete loops (e.g. black lines in Fig. 3B, E and G). Especially the initial part of the trajectory was largely influenced by the start orientation. For example, in trials with the visual pattern located posteriorly, animals walked several steps away from the pattern before eventually turning towards it (e.g., see Fig. 3B, D, F). Nevertheless, animals approached the pattern in more than 80% of trials if a high-contrast edge was present (e.g. patterns *Bar* and *Edge* in Fig. 3B, C; see also box plots in Fig. 4A, B), suggesting robust visual orientation behaviour even if the pattern was located behind them. The probability to be *on target* was decreased if no high-contrast edge was present (see box plots in Fig. 4A, B, E of cohort 1; Friedman’s test for matched samples, *p* = 0.002; Bonferroni-corrected Wilcoxon’s signed rank post-hoc tests, *p* = 0.0078) and reduced to chance level for the wide Gaussian pattern without edges (box plot in Fig. 4F) and the *Edge20* pattern with contrast below 50% (box plot in Fig. 4D).

To compare the distributions of viewing direction during approach (histograms below visual stimuli in Fig. 4) to distributions of head positions at the arena wall (circular histograms in Fig. 4), we fitted both unimodal and bimodal Gaussian mixture models (GMMs) to each one of these distributions (Suppl. Mat. S3-A). The results show that head positions at the arena wall were better described by a bimodal GMM whenever the visual pattern had two contrast edges (Bar, Edge20, Edge50; A, C, D in Fig. 4 and Suppl. Mat. S3-A), whereas unimodal GMMs provided better fits for two of three patterns with a single or no contrast edge (Edge, Gauss180; B, F in Fig. 4 and Suppl. Mat. S3-A). The result for the *Gauss90* pattern was intermediate, with a slightly better fit for the bimodal GMM. Here, the peaks of the bimodal GMM were close to the regions of highest contrast though with a bias towards the centre of the pattern. In case of the *Bar* pattern, the circular histogram in Fig. 4A has two peaks, one on either side of the bar (with AIC of the bimodal GMM being much lower than that of the unimodal GMM; Suppl. Table S3-A2). Similarly, though with less pronounced peaks, circular distributions for the *Edge50*,* Edge20* and *Gauss90* patterns (Fig. 4C, E, D) were better described by bimodal distributions (bimodal AIC < unimodal AIC in Suppl. Table S3-A2). We conclude that the position at which animals reached the wall was best predicted by the location of high-contrast regions or, in case of the wider Gaussian pattern, by the “centre of darkness”.

This was different for viewing directions during the approach. For several patterns the distribution of viewing directions (rectangular histograms in Fig. 4) differed from the distribution of head positions. This was particularly evident for the *Bar* pattern, where viewing directions showed a unimodal distribution (Fig. 4A with single peak below bar; lower AIC for the unimodal GMM; Suppl. Table S3-A2). In comparison, viewing distributions for approaches of the *Edge20* and *Edge50* patterns were bimodal (Fig. 4D, E and Suppl. Mat. S3 A-1), with a slight asymmetry in favour of the darker edge (Suppl. Fig. S3 A-1: larger λ in C, positive bias of both µ in E). When approaching a Gaussian pattern, viewing directions appeared unimodal (Fig. 4E, F) though skewed in case of the wider *Gauss180*. The more skewed distribution of the *Gauss180* pattern was also better described by a bimodal GMM (Suppl. Table S3-A2). We conclude that the most likely viewing direction during the approach may not coincide with the most likely position of arrival at the arena wall.

Differences in the approach behaviour, including the likelihood of *on-target* arrivals at the wall could be explained neither by differences in walked distance nor by differences in walking speed (Suppl. Mat. S3 B). However, the overall curvature of a path was increased for the *Edge20* pattern (*p* = 0.0468 after Bonferroni correction) indicating that the animals walked multiple curves during these trials. Indeed, three of the eight trials in Fig. 3E comprise loops. Additionally, the probability to be *on target* was reduced to chance level for this pattern, further indicating that the animals were not strongly attracted by any visual feature of this pattern (Fig. 4D).

We conclude that target choice may occur late in a trial. Whereas low luminance appears to determine initial heading direction and viewing direction throughout the approach, the location of high-contrast edges predicts the location at which the animal arrives at the wall. In the absence of high-contrast edges animals reach the wall where they would be expected to aim for if guided by a combination of negative phototaxis and edge orientation.

### Critical edge contrast can be titrated against a luminance-modulated distractor image

**Fig. 5:**
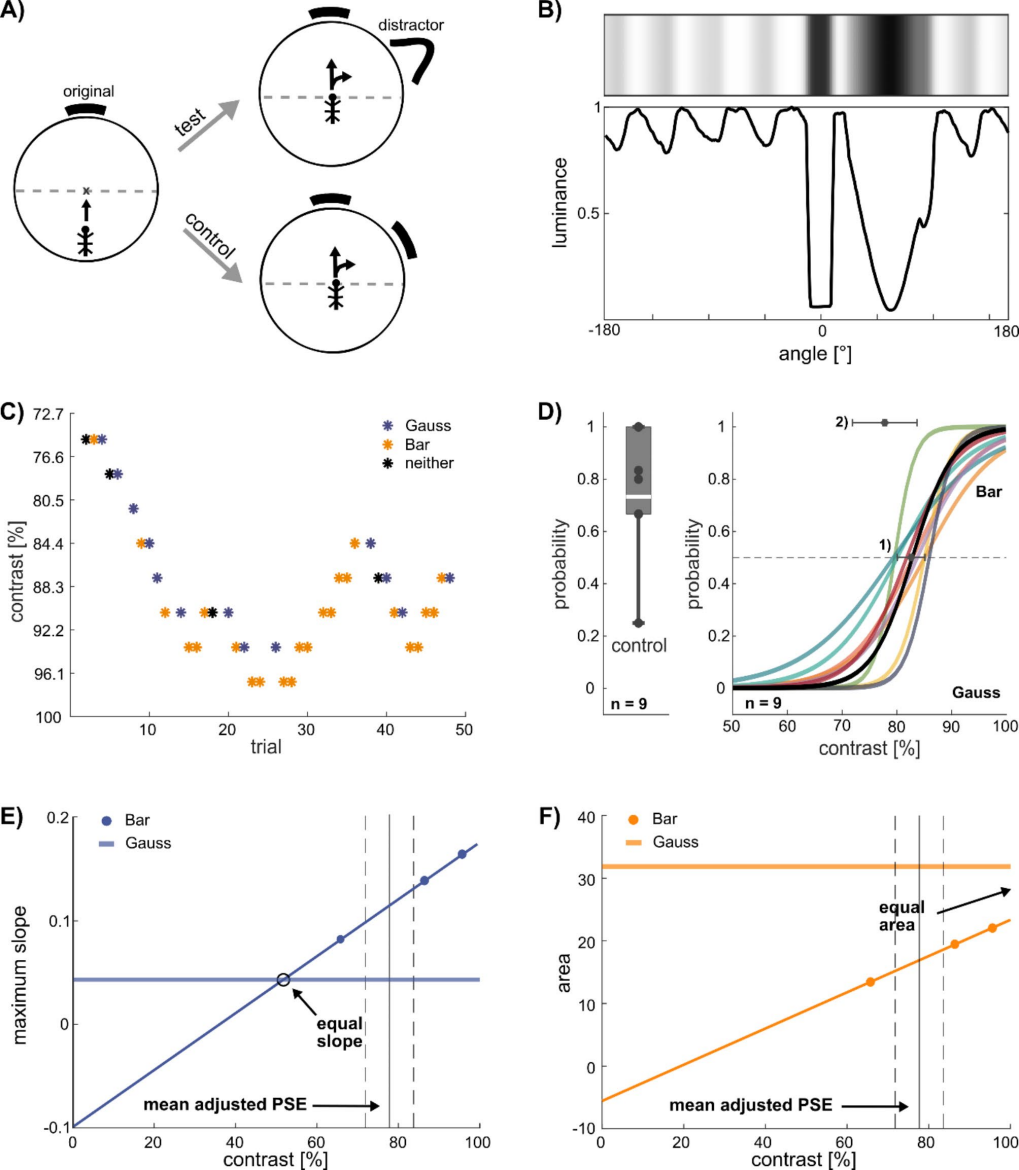
Both luminance and contrast determine the attractiveness of visual targets. (**A**) Two-alternative choice paradigm. The side of the distractor pattern was randomized. (**B**) Smoothed image of the arena screen (top) with corresponding normalized luminance measurement. Patterns are described by angles as seen from the arena centre. Note that periodic luminance modulations remained despite improved soft edge blending compared to experiment 2. (**C**) Exemplar staircase of one animal. (**D**) Boxplot shows the probability of an animal to choose the original pattern over the distractor pattern in control trials. Coloured lines show probability of choosing the original *Bar* pattern per animal. Horizontal dashed line shows 50% probability. Numbered error bars show two different choice thresholds (see Material and Methods): (1) Contrast at which animal chose the original as frequently as the distractor (*Point of Subjective Equality*, PSE); (2) Contrast at which animals chose the distractor with the same choice probability as in controls (adjusted PSE). A high probability corresponds to a choice of the Bar pattern, a low probability indicates that the Gaussian pattern was chosen. (**E**) Slope of steepest luminance gradient as a measure of local contrast. Dots indicate measured gradients of three *Bar* pattern of varying contrast. Measured values were used to fit a linear function. Black circle shows the intersection of both lines, i.e. the contrast at which the maximum gradient of both patterns would be equal. Black vertical line shows the measured mean adjusted PSE from (D), with dashed lines indicating the standard deviation of the adjusted PSE. (**F**) Area below curve B, as a measure of mean luminance. Same graph details as in (E). Black arrow points towards the hypothetical intersection of both lines, highlighting the contrast at which the mean luminance of both patterns would be equal.

The previous experiment revealed that stick insects strongly prefer regions of high contrast (e.g., edges) over areas of low luminance (e.g. in *Bar* pattern of Fig. 4A). However, we also found that final positions were biased towards the darker area of contrasted patterns (e.g. GMM results for *Bar* and *Gauss90* patterns of Suppl. Mat. S3 A, E, see also Fig. 1D; Table 2). Since the former suggests the presence of an edge orientation mechanism whereas the latter preference would be better described by negative phototaxis, we wanted to assess the relative contribution of these two mechanisms. To do so, we designed a two-alternative choice paradigm and used an adaptive staircase method to titrate the edge contrast of an attractive *Bar* pattern against a fixed, luminance-modulated Gaussian pattern without sharp contrast edges. For this, the contrast of the *Bar* pattern could be lowered by increasing the luminance of the *Bar*. The objective of this experiment was to determine the *Point of Subjective Equality*, PSE, at which the original target pattern *(Bar*) was equally attractive to a Gaussian distractor pattern (Fig. 5A, B). Our rationale was that, if edge orientation alone dominated target choice, the PSE should be reached if the “maximum local contrast” of the *Bar* and the Gaussian were equal. If, however, phototaxis alone dominated target choice, the PSE should be reached if the mean luminance of both patterns were equal. Since the “overall contrast” of the Gaussian, i.e. the difference in luminance between its brightest and darkest spot, was always larger than that of the *Bar*, a contrast-dependent change in preference for the *Bar* could only depend on local contrast. Given the fact that the spatial resolution of eyes is diffraction-limited (e.g^48^). , and the acceptance angles of insect ommatidia may exceed a few degrees^49^, the optical filter properties of the compound eye had to be taken into account to estimate the maximum gradient of luminance seen by the retina. Therefore, we convolved the measured luminance of the visual pattern with a Gaussian kernel equivalent to the acceptance angle of the stick insect compound eye^30^ and determined the maximum slope. Though different in rationale, this corresponds to the use of the maximum gradient as a signal of edge location in human psychophysics^50^. To test whether phototaxis alone could explain the PSE, we related the PSE to a measure of mean luminance. This was calculated as the area beneath the convolved luminance curve.

Our experiments showed that orientation behaviour towards these luminance patterns was robust enough to be tested for up to 50 trials per animal, with only few trials in which the animal did not decide for either target. Control trials in which the distractor was identical to the original *Bar* pattern, showed that animals continued to walk towards the original target. This suggested that animals preferred to not change their walking direction (Box plot in Fig. 5D). This bias was taken into account by adjusting the PSE (usually at 50%). The adjusted PSE was the contrast at which the *Gauss* pattern achieved the same choice rate as the distractor *Bar* during control trials (for details see Material and Methods section). Test trials started with a relatively low edge contrast of the *Bar* (< 75%). Depending on whether or not the animal was distracted by the *Gauss* pattern, the edge contrast was increased or decreased, respectively (see contrast staircase in Fig. 5C). The results show that the mean adjusted PSE of *N* = 9 animals was at an approximate edge contrast of 77.8% (Fig. 5D). For comparison, measured contrast of three *Bar* patterns predicted that, for edge orientation alone (i.e. max. luminance slope), the PSE should have laid at an edge contrast of 51%, considerably below the titrated PSE (see intersection of the two lines in Fig. 5E). On the other hand, negative phototaxis (i.e. area under luminance modulation) predicted the PSE for an edge contrast of 130%, well beyond the possible range (extrapolated intersection in Fig. 5F). By comparing the relative distances of these predicted values from the experimentally measured one, we conclude that mechanisms underlying edge orientation have a larger influence on visual target choice than do mechanisms underlying negative phototaxis, with a ratio of approximately 2:1. Nevertheless, neither edge orientation nor phototactic mechanisms alone, could explain the experimentally determined PSE, further substantiating the findings of the previous experiments.

## Discussion

The aim of our study was to systematically investigate the relative significance of static image luminance and contrast in visual target choice and visually-induced turning in freely walking stick insects. We found that visual contrast dominates visual target choice, though with a bias towards stimulus regions with low luminance (Figs. 1 and 2). Additionally, we showed that the direction of heading during trials may differ from the terminal positions at the arena wall (Fig. 4), with direction of heading being affected much less by high-contrast image features than terminal positions. Finally, direct estimates of the adjusted point of subjective equality of contrast and luminance (Fig. 5) suggest that mechanisms underlying contrast-dependent edge orientation have a stronger influence on visually induced turning than do mechanisms underlying luminance dependent phototaxis, with an approximate ratio of 2:1.

### Cues in visual orientation behaviour: Position versus motion, luminance versus contrast

Our experiments exploit the fact that stick insects, like many – if not all – walking insects show a natural, robust tendency to approach selected static visual targets. The large open field arena surrounded with luminance-modulated 360° images offered the possibility to investigate approach behaviour in unrestrained walking animals, with natural coupling of approach and the self-induced change in visual target size and location. Our results confirm earlier findings that stick insects show reliable choice preferences for static visual objects^29,32^ and reliably approach dark objects with high-contrast edges^15,33,34^.

Early studies on visual orientation behaviour in insects distinguished a position- and a motion-sensitive component^10,51,52^. The position-sensitive component allows to track and approach a particular image feature such as a black-and-white edge by turning towards the feature, so as to shift its position on the retina from a lateral to a medial position. The motion-sensitive component counteracts this response by turning to minimise the velocity between itself and the image feature. While our current experiments acquired an estimate of the combined position- and motion-sensitive response (viewing direction, i.e., head orientation, across a whole trial), our setup does not allow the separate investigation of these responses as it is possible in tethered locomotion (e.g^11^), or in a virtual reality setup (e.g^3^). As walking insects can infer distances from motion parallax during translational movement^3^, self-induced changes in perceived height of the arena wall or motion differences between the edges of the single dark target may have provided additional cues for orientation. To assess the extent to which such motion cues may have supported or rather counteracted the described effects of static image cues, future modelling studies may determine the self-induced change in visual image geometry based on experimental measurements as ours, and probe the relative weight of either cue type. In particular, it would be interesting to test whether self-induced motion cues can contribute to the qualitative differences between viewing angle distributions and final position in Fig. 4.

Our experimental setup was designed to investigate behavioural responses to monochrome visual patterns of equal distance, height and with one-dimensional (horizontal) variation of luminance only. For testing the relative significance of static image luminance and its spatial derivative, i.e., contrast, we systematically varied target width, contrast, background luminance and/or the number of edges per target. While our results clearly show that contrast weighs stronger than luminance alone (Figs. 1D, 2C and 5), a number of observations reveal that luminance modulation alone causes a robust and reliable effect, too. First, the orientation towards identical contrast edges may differ, depending on the luminance of the areas between them (Fig. 1C). Second, animals that did not approach contrast edges clearly showed a preference for darker regions of the luminance-modulated background (Fig. 2B). Third, the edge contrast at which stick insects show equal preference for a Gaussian pattern is considerably higher than expected from the shallow contrast gradient of the Gaussian (Fig. 5C-E), indicating that another parameter of the Gaussian pattern counter-balances this difference (Fig. 5F). This is similar to results on tethered walking moths^18^ that show a clear preference for contrast edges (if present) but head for the darkest stimulus region (rather than the steepest luminance gradient) if no contrast edges are present. In fruit flies, visual orientation behaviour is strongly affected by the physiological state of the animal: Whereas *D. melanogaster* with intact wings exhibit positive phototaxis^6^ wingless adults (either with clipped wings or mutant flies) show negative phototaxis (e.g^4,6^. In nymphs of the Australian stick insect *Extatosoma tiaratum*, the “sign” of phototaxis may also switch, though in dependence of age and only in case of high mean luminance^15^. Whether the luminance-dependent response observed in our study might also change throughout development remains to be tested in future studies.

### Target choice and visually induced turning: early versus late response components

As *C. morosus* is an obligatory walking insect, our results refer to the animal’s natural (and only) mode of locomotion in its intact physiological state and under closed-loop stimulus-response conditions. Similar to^4^, the free-walking condition allowed us to compare different phases of the approach towards static objects (Figs. 3 and 5). The common view that visual orientation towards static targets is governed by feedback control both in flight^10^ and in walking^14,17^ is supported by our observation that late parts of the approach trajectories tended to be fairly straight (Fig. 3). However, this view is challenged by the observation that early parts of the walking trajectories sometimes comprised multiple loops (Fig. 3), proving that the decision to approach a certain feature did not always happen right at the start of a trial. To address the question whether the target choice that determines subsequent feedback-controlled approach behaviour occurs early on during a trial (and only once) or may occur later (and potentially multiple times) during the approach, Fig. 4 relates the viewing direction throughout the approach to the final positions at the arena wall. The result is similar to that reported for flies and locusts in a two-choice orientation task^1^, in which animals initially move in the “average target direction”, i.e. towards the centre between the targets, until a critical angular difference is reached. At this point they randomly selected either option and continued moving towards the chosen object. In case of the symmetrical target with edges (the 90° Bar), Fig. 4A reveals that the viewing direction throughout the trials peaks near the centre of the 90° Bar, whereas the final head positions cluster at its edges. This indicates a switch of heading direction, with the early approach phase heading towards the centre, followed by a late turn towards one of the edges.

Moreover, the spread of the final head positions around either edge in Fig. 4A can explain the width-dependent transition from unimodal to bimodal distributions between the 22.5° and 45° bars in Fig. 1D. Tethered walking mealworm beetles (*Tenebrio molitor*^17^), and gypsy moths (*Lymantria dispar*^18^), show the same width-dependent “smearing” of heading distributions – at least for pooled data - with “edge fixation” for wide bars and “centre fixation” for narrow bars. Preiss and Kramer^18^ further showed that the heading distributions of individual moths sometimes showed a peak at the centre of a 90° bar, similar to our pooled distribution for freely walking stick insects (Fig. 4A). In agreement with both of these results, we propose that during the initial approach of a 90° bar - and throughout the entire trial in tethered walking moths -, the centre of darkness and the centre of both high-contrasted edges are located in the same direction. As a consequence, both the luminance-dependent and the contrast-dependent response components result in the same heading. As the animal moves toward the target, the angular distance between the two edges increases, assumingly approaching a tipping point as proposed by^1^, at which the animal would randomly choose either edge. As our results in conjunction with Fig. 5 indicate a stronger weighing of contrast than luminance, only few animals reach the wall at the centre of the bar.

An alternative explanation could be a distance- or time-dependent change in the relative weight of the competing cues luminance and contrast, as suggested in a navigation model with multi-cue integration^53^. In this case, the luminance-dependent response component would dominate the heading during the initial phase of the approach, whereas the contrast-dependent response (e.g. edge fixation) would determine the final position.

As our experiments were not designed to tell the difference between the two scenarios sketched above, future experiments could do so with appropriately designed two-target situations and careful comparison of measured heading time courses with predicted decision points. So far, either scenario can explain the discrepancy between viewing distribution and final position, along with a fairly late turns towards one of the edges, but neither of them can explain the initial loops observed in Fig. 3.

### Luminance and contrast: Distinct cues driving distinct behaviours?

**Fig. 6:**
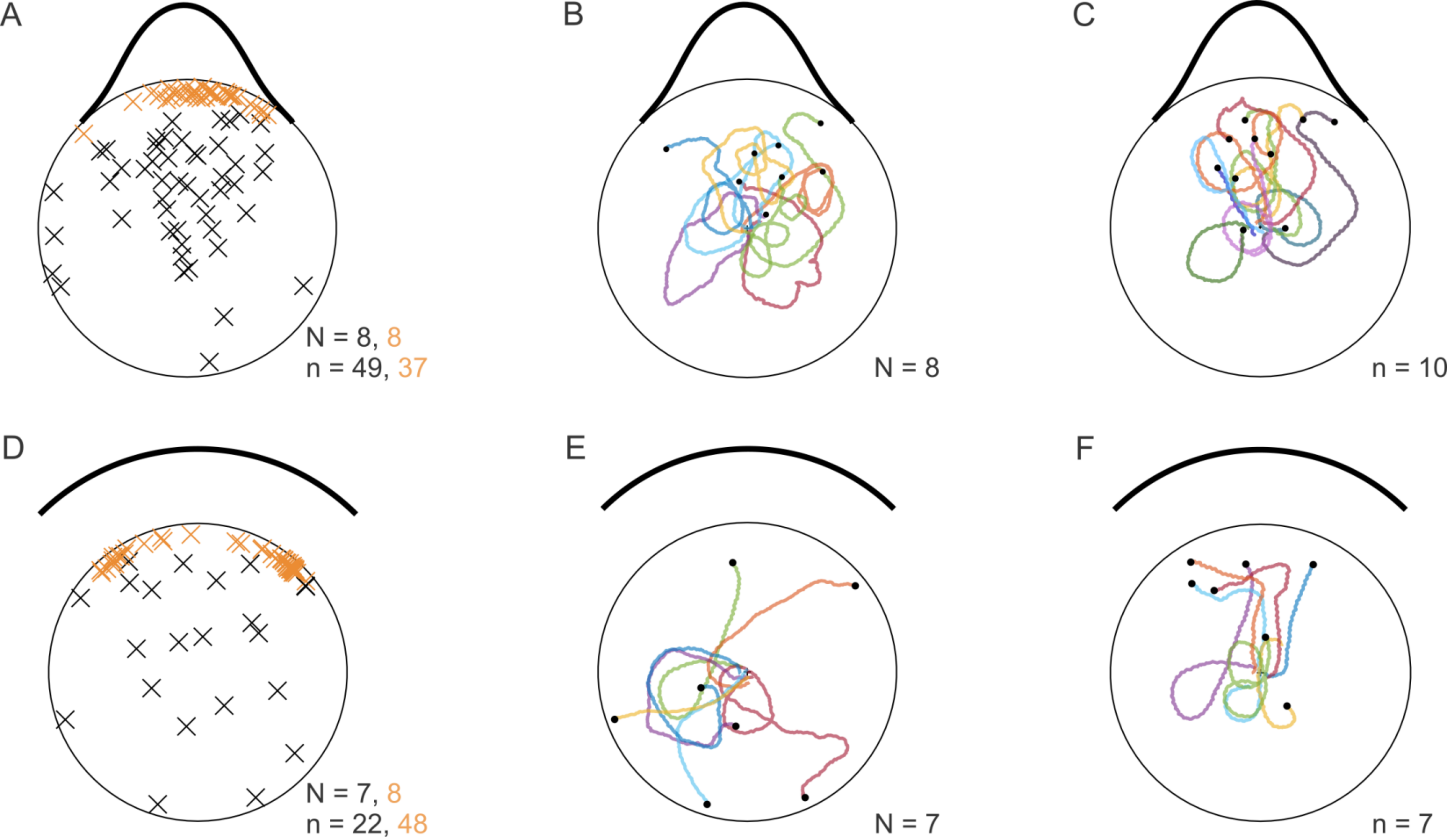
Comparison of trajectories of trials in which animals were ‘off target’ for Gaussian and Bar patterns. (**A**,** D**) Final head positions for ‘*on target*’ (orange) and ‘*off target*’ (black) trials of N animals in a total of n trials. Animals that were ‘*off target’* showed a tendency to approach the region subtended by the Gaussian pattern, as revealed by a non-uniform distribution of final positions (black crosses) for the Gaussian pattern (A) but a random distribution in D. (**B**,** E**) For illustration of different overall walking behaviour, coloured lines show the prothorax (T1) trajectories of one randomly selected ‘*off-target*’ trajectory per animal for the Gaussian and Bar pattern. (**C**,** F**) For illustration of persistent differences per animal, coloured lines show the prothorax (T1) trajectories of all ‘*off-target’* trajectories for one animal for the Gaussian and Bar pattern.

In his introduction to what he considered to be a new kind of orientation behaviour, Kalmus^33^ observed that *C. morosus* nymphs not only orient themselves towards black and white edges, but also climb upon the visual pattern to align their body axis with the contrast edge. He concluded that this alignment behaviour was too complex to be considered as simple taxis. Although we didn’t quantify the alignment of animals with contrast edges, our results show that animals reached the arena wall 27% points less often in case of the 90° Gaussian pattern than for the 90° Bar (50% vs. 77%, see Suppl. Mat. S5). Still, the fraction of trials in which animals climbed the wall was above 85% in both cases, irrespective of whether or not these trials ended up “on-target”, i.e. in the pattern range (Suppl. Mat. S5). A closer look at the trials in which animals did not reach the wall revealed an overall difference in walking behaviour: When confronted with a Gaussian pattern, animals tended to stop walking in front of the dark part of the wall, or within the sector subtended by the luminance-modulated pattern (Fig. 6A-C). Accordingly, the distribution of end positions of these walking paths had a statistically significant directional bias with a mean direction of 9° (Rayleigh test, *p* < 0.001, *n* = 49). To the contrary, when the same animals were confronted with the Bar pattern, they tended to reach and – as described by Kalmus - climb the wall near the contrast edge (Fig. 6D). In those trials that stopped before reaching the wall (Fig. 6E, F), the distribution of end positions had no preferred direction (Rayleigh test, *p* = 0.26, *n* = 22).

Stick insects are known to turn towards and reach for objects that they touch with their antennae^20,22^. Moreover, they readily sample walls and edges with their antennae^21^ and shortening the antennae delays the climbing as a result of later physical contact with the obstacle^25^. In agreement with this, animals that reached the wall tended to climb it, irrespective of what stimulus had been presented and even when they were off target (Suppl. Mat. S5). Given the differences in approach behaviours for static visual images with and without high-contrast edges (Figs. 5A and E and 6A and D) we propose the superposition of two distinct, visually driven orientation behaviours: A luminance-related, negative phototaxis behaviour that governs the preference for darker, potentially sheltered regions and a contrast-related edge orientation behaviour – or photohorotaxis sensu Kalmus – that governs the approach and subsequent climb of an object that, in nature, could be characterised by a visual contrast edge. The associated distinction in behavioural function would correspond well with an early, luminance-dependent orientation towards dark regions and a later, contrast-dependent landmark approach.

### Outlook

Our data suggest the presence of two distinct components of visual orientation behaviour in walking stick insects. This distinction is grounded on the observations that (i) luminance and its spatial derivative – contrast – differentially affect visual target choice; (ii) differences between viewing direction and final position are in agreement with an early, potentially luminance-dominated phase and a late, contrast-dominated phase of target approach; and (iii) approaches of high-contrast targets are accompanied by high likelihoods of climbing the target. Although little is known about the visual system of stick insects, work on other insects has shown distinct visual pathways to encode contrast and luminance^35^, leading to the suggestion that luminance modulates contrast-dependent visual behaviours. Since the stick insect *C. morosus* is an established study organism in sensorimotor control, the present study sets the stage for a systematic investigation of leg movements and inter-leg coordination in visually induced turning of unrestrained walking stick insects. In particular, our results call for a test whether the two distinct components of visual orientation behaviour are mirrored and/or supported by distinct likelihoods of particular step types (e.g. as in climbing^23^), or distinct changes in inter-leg coordination^28^.

## Electronic supplementary material

Below is the link to the electronic supplementary material.

Supplementary Material 1

## Data Availability

Experimental data are available on the research data repository of Bielefeld University at https://pub.uni-bielefeld.de/record/3002160.
