# Supplementary material for: Contrast and luminance dependence of target choice and visual orientation in walking stick insects
Source: Sci Rep. 2025 Apr 10;15:12226. doi: 10.1038/s41598-025-90650-8 (PMC11986023; doi:10.1038/s41598-025-90650-8)
Supplement: Supplementary file 1 — Supplementary Material 1 [file 41598_2025_90650_MOESM1_ESM.pdf]

S1: Preliminary experiments on detectability and target choice

Preliminary experiments were conducted to identify a suitable "standard bar width". To do so, we assessed the reliability of visual target choice for different landmark sizes, the walls of the open-field arena were marked with three black vertical bars (cardboard stripes) of variable width (1.25°, 2.5°, 5° and 10°). Bars were placed in the middle of sectors 30°, 150° and -90°. The arena was diffusely illuminated by standard laboratory lights (no natural light sources, such as windows). Mean luminance of the arena wall was I_{max} = 250 cd/m^2, that of the bars was I_{min} = 8 cd/m^2, as measured by a Minolta Luminance Meter 1°. This resulted in a Michelson Contrast of $C = (I_{max} - I_{min}) / (I_{max} + I_{min})$ = 95 %. Trials were evaluated as explained for experiment 1 in the main manuscript, except that the chance level of the control trials was 3/36, or 8.3 % (because of three black bars, instead of one).

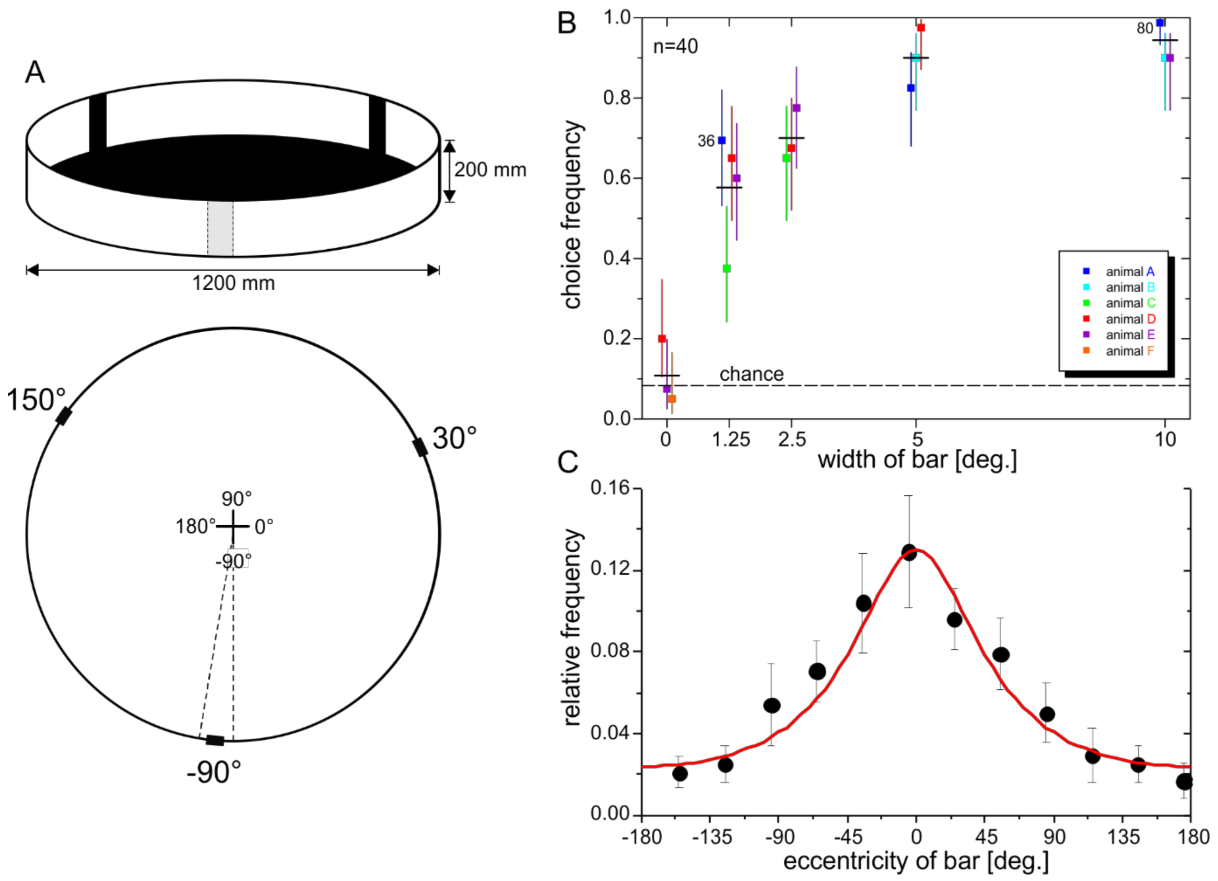

Supplementary Figure S1: Choice frequency for black bars depends on visual size of the bar. *A) Animals walked in an arena equipped with three black bars spaced by 120°. B) The width of the bars was varied. Choice frequency is the fraction of walks toward one of the three target sectors with a black bar. Symbols and error bars show choice frequency and 95% confidence intervals for single animals. Different colours stand for different animals. Trial number per animal and bar width was 40, except for two cases for which the numbers are indicated next to the symbol. Bar width zero is a control with no bars in the target sectors. Chance level was 1/12, as marked by the broken line. Horizontal line segments show choice frequency of pooled trials from three to four animals. C) Relative frequency of selected landmarks (here: width 2,5°) depends on eccentricity at trial onset. Animals have a preference for central landmarks but do choose peripheral ones despite the presence of more central ones. Red line shows wrapped Cauchy distribution fitted to the data using the software Origin (MicroCal).*

Supplementary Material to Meschenmoser and Dürr (2025): *Contrast and luminance dependence of target choice and visual orientation in walking stick insects.*

Since Jander und Volk-Heinrichs [1] reported reliable orientation towards vertical black bars on white background, we started out with this type of landmark and tested the effect of bar width on reliability of target choice. To do so, we exposed animals to three equidistant vertical black bars (Fig. 1A) and determined the choice frequency, i.e., the fraction of trials in which the animals walked towards a sector of the arena wall that contained one of these landmarks. Mean choice frequencies were ≥ 90 % for bar widths of 5° or 10°, declined to less than 60 % at 1.15° and reached chance level for a blank arena (controls; Fig. 1B). Given the high reliability of the 10° bar and the fact that this width is larger than the visual acceptance angle of both light- and dark-adapted, adult *C. morosus* [2], we opted for the 10° bar as the reference stimulus.

Since we estimated the visual field size of *C. morosus* to be at least ±150° of azimuth [1], we assumed that animals would be able to see all three landmarks at the start of a trial, unless one landmark was straight behind them. Moreover, at least one landmark would be seen at ≤ 60° azimuth relative to the midline, suggesting that animals would never have to turn more than 60° when choosing the landmark with the smallest eccentricity (visual azimuth at trial onset). To illustrate how the placement of a landmark within the visual field affects its choice likelihood, Fig. 1C plots the relative frequency of a chosen landmark against its eccentricity at trial onset. The resulting distribution is nearly mirror-symmetrical and bell-shaped, with no zero values. This shows that stick insects clearly favour more central landmarks over more peripheral ones, though even very peripheral landmarks get chosen occasionally. The wrapped distribution in Fig. 1C can be fitted with a circular distribution with large spread, in this case a zero-centred, wrapped Cauchy distribution with $p = 0.4$.

S2: Supplement to experiment 1: Comparison of Michelson Contrast and Weber Contrast

Except for Fig. 1C of experiment 1 A, the usage of the contrast measures Michelson Contrast, C_M, and Weber Contrast, C_W, does not affect any conclusions, mainly because the measures differ only with regard to scaling and normalisation. Applied to the visual stimuli used in this paper, they are calculated as

$C_M = (L_{max} - L_{min})/(L_{max} + L_{min})$,

where L_{max} and L_{min} are the maximum and minimum luminance values of the arena projection, and

$C_W = |L_{BG} – L_{FG}|/L_{ADAPT}$,

where L_{BG} and L_{FG} are the luminances of the background and foreground, respectively, and L_{ADAPT} is the mean luminance of the arena projection. For example, for our standard stimulus of a black bar of 10° width on a uniform white background (350° width), L_{ADAPT} is $L_{BG} \cdot 35/36 + L_{FG}/36$.

The expectations for part A of experiment 1 differ for the two contrast measures in that C_W - dependent choice rates should be much higher for a white bar on black background ($C_W = 30.57$) than for a black bar on a white background ($C_W = 1.023$), whereas they should be equal for C_M. In fact, the results of Fig. 1C show that choice rates for the white bar are much lower than for the black bar.

The finding that hit rates depend on image contrast (Fig. 2C) is independent of which contrast measure is used (Suppl. Fig. S2), because the different denominators only change the scaling of the abscissa. Multiple regression of hit rate against bar luminance and contrast were almost equal for either contrast measure, with $r^2=0.729$ for C_M and $r^2=0.725$ for C_W.

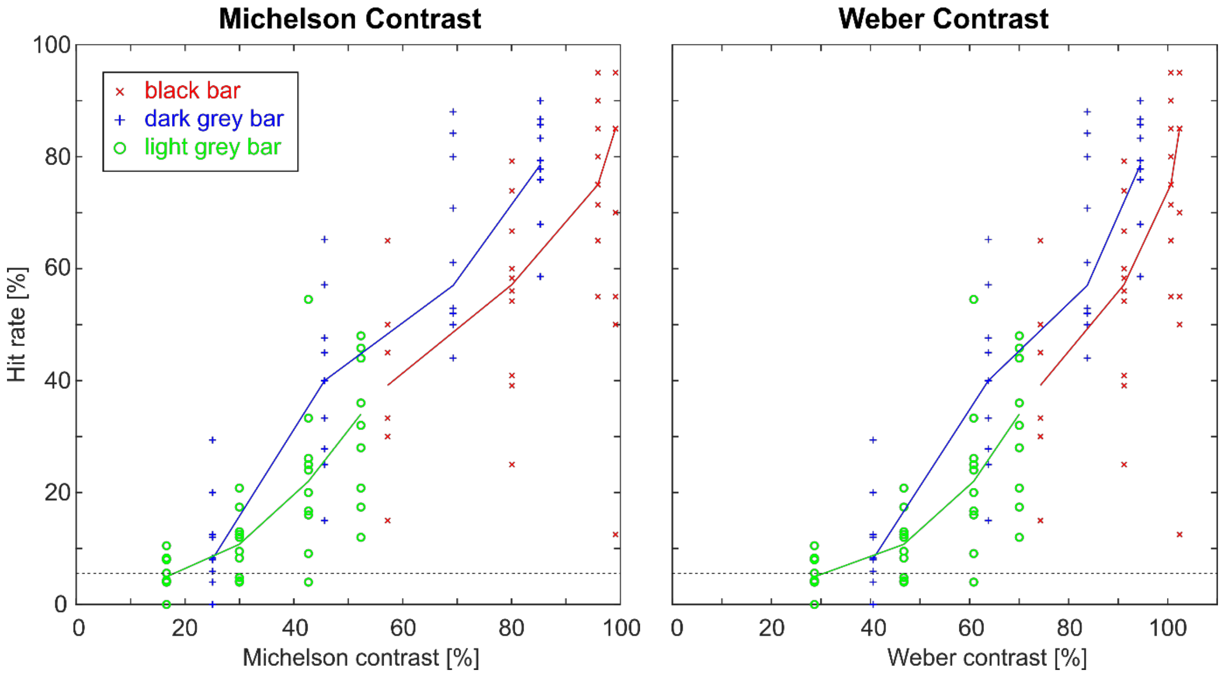

Supplementary Figure S2: Comparison of contrast dependencies for Michelson and Weber contrasts. *Left: Same graph as in Fig. 2C. Right: Same data, but plotted against Weber contrast. The median choice rate curves appear slightly skewed, but the overall finding that choice frequency consistently depends on contrast is the same as for Michelson contrast.*

S3: Supplement to experiment 2:

S3 A: Comparison of viewing direction and final position for experiment 2

Suppl. Fig. S3-A1 complements Fig. 4 of the main manuscript, relating the positions of arrival at the arena wall to the distribution of viewing directions throughout the approach. Note that Suppl. Fig. S3-A1 includes all n_W trials that terminated at the wall, whereas Fig. 4 includes the n_T *on-target* trials only. In both figure variants, the two distributions per panel differ strongly with regard to sample size and statistical independence of data points. The head positions are independent, with a single data point per trial. In contrast, viewing directions are time series data, with dependent samples per trial and a much larger sample size. Moreover, distributions of viewing directions are broadened by rhythmic yaw movements of the stick insects' head and fluctuations in walking direction, e.g., due to step-to-step variation. Lastly, the effect of head movements on viewing direction depends on the distance to the arena wall.

To compare viewing direction and final head position distributions, we fitted two kinds of Gaussian mixture models (GMM) to each distribution: (i) A unimodal Gaussian mixture model consisting of two identical Gaussians, i.e. with same means and standard deviations. As prior estimates we used a mean of 0° and a standard deviation of 20°. This was compared with (ii) a bimodal GMM that comprised two Gaussians for which the standard deviation were kept the same but the means were allowed to differ. As prior estimates we used the borders of the visual patterns for the means and 20° for the standard deviation. In bimodal GMMs, Gaussian components were scaled with a factor lambda, with the two lambdas adding up to one (in unimodal GMMs, both lambdas were 0.5). The GMM parameters of the model fits shown in Suppl. Fig. S3-A1 are tabulated in Suppl. Tab S3.-A2.

The comparison of unimodal and bimodal GMMs yielded the same type of distribution for viewing direction and final head position for three (*Edge*, *Edge20, Edge50*) out of six patterns. Of these, the *Edge20* pattern was special in that both distributions were better explained by a bimodal GMM, though the mean of the component 2 was very different: for the viewing direction, it was not located near the lighter edge (mu = -0.41, left edge in in Fig. S3-A1), whereas it was for the head position (mu = -52.25).

For the *Bar* and *Gauss90* patterns, GMM fits favour a unimodal distribution for the viewing direction but a bimodal distribution for the head position. For the wider Gaussian pattern (*Gauss180*) GMM fits favour a bimodal distribution for the viewing direction with both means (mu_{Left} = -26.56, mu_{Right} = 23.41) close to the steepest changes in luminance at ±30 degrees, whereas the head position distribution was described better by the unimodal fit.

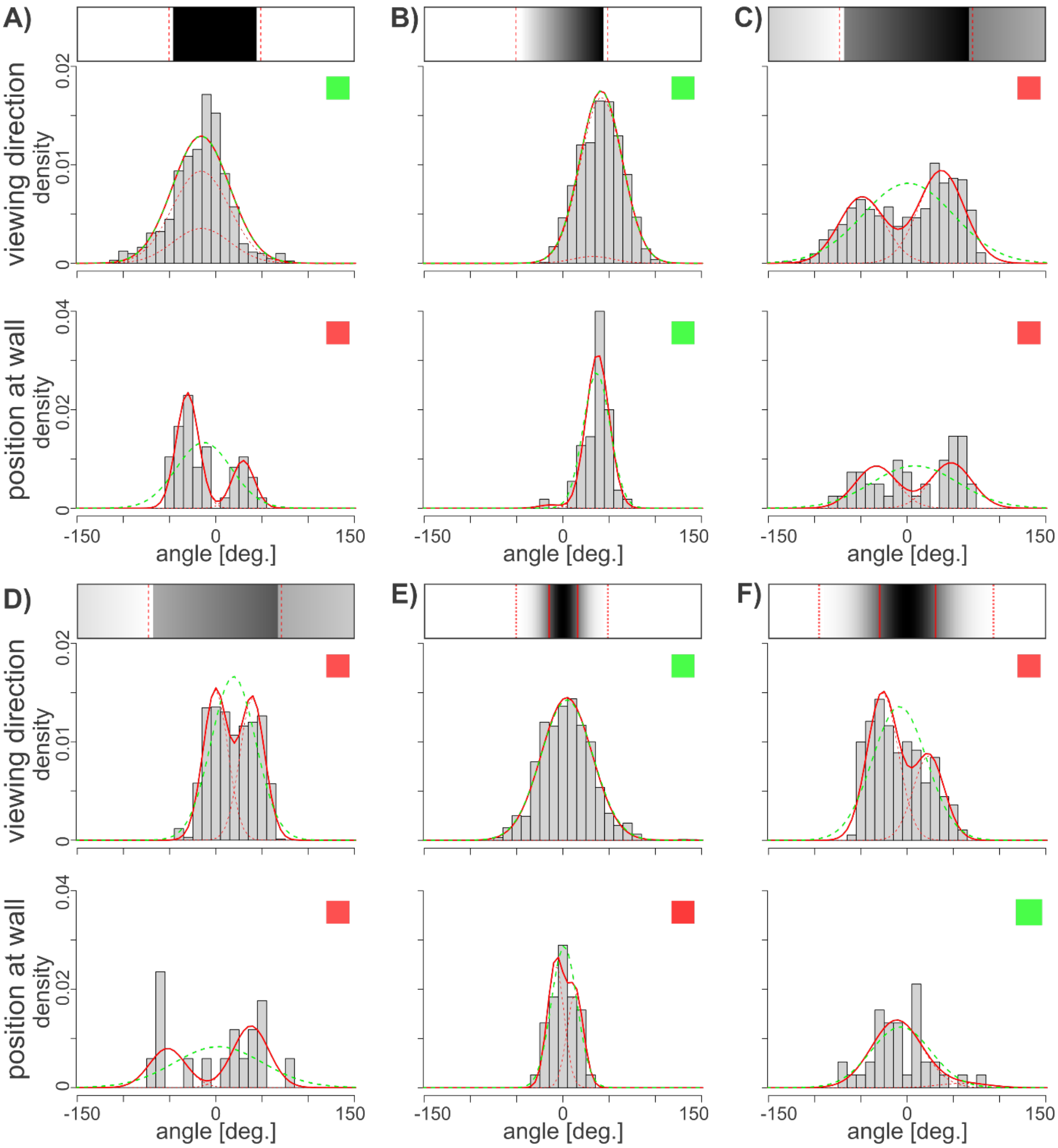

Supplementary Figure S3-A1: Positions of arrival at wall can differ from viewing direction during the approach

Histograms (bin width = 10 deg.) below visual patterns show the median probability distributions of viewing direction per animal (N = 8) for at-wall trials. Red solid lines show Gaussian mixture models (GMM) after fitting two Gaussians with in the same standard deviation but independent means, thus allowing for bimodality. For each two-component GMM, red dashed lines show the Gaussian components. Green dashed lines show GMM after fitting two Gaussians witch the same standard deviations and means, thus forcing the fit of a unimodal distribution. Coloured squares indicate which model achieved the better fit (red = bimodal, green = unimodal, for details see Suppl. Table S3-A2). Histograms (bin width = 10 degrees) at the bottom of each panel show the head positions at the time of arrival at the arena wall, with same graph details as in viewing direction subplots.

Supplementary Table S3-A2: GMM parameters and evaluation

Pattern	Response	Unimodal	Bimodal
**Bar**	**Viewing**	comp 1 & 2<br>lambda 0.5<br>mu -15.9043<br>sigma 30.8343<br>loglik -23656.12<br>AIC: 47318.25 (3 est.)<br>χ^2: 515.48, df:20	comp 1 comp 2<br>lambda 0.7255 0.2745<br>mu -16.0729 -15.4588<br>sigma 30.8331 30.8331<br>loglik -23656.12<br>AIC: 47320.25 (4 est.)<br>χ^2: 515.48, df:20
	**Position**	comp 1 & 2<br>lambda 0.5<br>mu -12.9792<br>sigma 29.8869<br>loglik -231.1853<br>AIC: 468.3705 (3 est.)<br>χ^2: 26.961, df:9	comp 1 comp 2<br>lambda 0.7076 0.2924<br>mu -30.5727 29.5992<br>sigma 12.0051 12.0051<br>loglik -216.0823<br>AIC: 440.1647 (4 est.)<br>χ^2: 6.3964, df:9
**Edge**	**Viewing**	comp 1 & 2<br>lambda 0.5<br>mu 41.4775<br>sigma 22.7505<br>loglik -20373.17<br>AIC: 40752.34 (3 est.)<br>χ^2: 136.68, df:12	comp 1 comp 2<br>lambda 0.0396* 0.9604<br>mu 32.5345 41.8464<br>sigma 22.6779 22.6779<br>loglik -20373.14<br>AIC: 40754.28 (4 est.)<br>χ^2: 136.97, df:12
	**Position**	comp 1 & 2<br>lambda 0.5<br>mu 36.6909<br>sigma 14.4107<br>loglik -224.78<br>AIC: 455.56 (3 est.)<br>χ^2: 9.939, df:7	comp 1 comp 2<br>lambda **0.0209*** 0.9791<br>mu -13.4158 37.7591<br>sigma 12.4156 12.4156<br>loglik -221.5022<br>AIC: 451.0045 (4 est.)<br>χ^2: 8.7047, df:7
**Edge 50**	**Viewing**	comp 1 & 2<br>lambda 0.5<br>mu 1.2697<br>sigma 49.0735<br>loglik -35023.71<br>AIC: 70053.43 (3 est.)<br>χ^2: 1624.1, df:21	comp 1 comp 2<br>lambda 0.4172 0.5828<br>mu -48.8862 37.1805<br>sigma 24.6388 24.6388<br>loglik -34234.82<br>AIC: 68477.64 (4 est.)<br>χ^2: 316.73, df:21
	**Position**	comp 1 & 2<br>lambda 0.5<br>mu 8.6829<br>sigma 46.2672<br>loglik -215.3883<br>AIC: 436.7765 (3 est.)<br>χ^2: 21.414, df:14	comp 1 comp 2<br>lambda 0.4813 0.5187<br>mu -33.3575 47.6915<br>sigma 22.3768 22.3768<br>loglik -209.4309<br>AIC: 426.8617 (4 est.)<br>χ^2: 11.641, df:14
**Edge 20**	**Viewing**	comp 1 & 2<br>lambda 0.5<br>mu 18.9843<br>sigma 23.9869<br>loglik -16055.38<br>AIC: 32116.76 (3 est.)<br>χ^2: 1571.8, df:11	comp 1 comp 2<br>lambda 0.5129 0.4871<br>mu **-0.4146*** 39.4092<br>sigma 13.3846 13.3846<br>loglik -15826.32<br>AIC: 31660.64 (4 est.)<br>χ^2: 503.1, df:11
	**Position**	comp 1 & 2<br>lambda 0.5<br>mu 2.8235<br>sigma 48.0419<br>loglik -89.9472<br>AIC: 185.8944 (3 est.)<br>χ^2: 5.2604, df:9	comp 1 comp 2<br>lambda 0.3889 0.6111<br>mu -52.2518 37.8710<br>sigma 19.4366 19.4366<br>loglik -85.17575<br>AIC: 178.3515 (4 est.)<br>χ^2: 4.4902, df:9

**Gauss 90**	**Viewing**	comp 1 & 2<br>lambda 0.5<br>mu 4.3235<br>sigma 27.9475<br>loglik -25840.75<br>AIC: 51687.5 (3 est.)<br>χ^2: 182.69, df:17	comp 1 comp 2<br>lambda 0.9981 **0.0019***<br>mu 4.0959 **125.87***<br>sigma 27.4481 27.4481<br>loglik -25808.94<br>AIC: 51625.87 (4 est.)<br>χ^2: 170.65, df:17
	**Position**	comp 1 & 2<br>lambda 0.5<br>mu 1.3158<br>sigma 13.7823<br>loglik -153.6084<br>AIC: 313.2168 (3 est.)<br>χ^2: 1.7897, df:6	comp 1 comp 2<br>lambda 0.5682 0.4318<br>mu -7.8710 13.4039<br>sigma 8.8827 8.8827<br>loglik -152.5727<br>AIC: 313.1453 (4 est.)<br>χ^2: 2.2867, df:6
**Gauss 180**	**Viewing**	comp 1 & 2<br>lambda 0.5<br>mu -8.3324<br>sigma 29.3602<br>loglik -25710.78<br>AIC: 51427.55 (3 est.)<br>χ^2: 1237.6, df:12	comp 1 comp 2<br>lambda 0.6352 0.3648<br>mu -26.5633 23.4105<br>sigma 16.8321 16.8321<br>loglik -25310.23<br>AIC: 50628.45 (4 est.)<br>χ^2: 548.63, df:12
	**Position**	comp 1 & 2<br>lambda 0.5<br>mu -6.8684<br>sigma 32.2867<br>loglik -185.9566<br>AIC: 377.9131 (3 est.)<br>χ^2: 1.251, df:13	comp 1 comp 2<br>lambda 0.9383 **0.0617***<br>mu -11.3246 60.8543<br>sigma 27.2148 27.2148<br>loglik -185.1739<br>AIC: 378.3478 (4 est.)<br>χ^2: 21.816, df:13

Model comparison: *Columns give fit parameters and goodness-of-fit estimates for the best unimodal (left) or bimodal (right) Gaussian mixture models (GMM). GMM parameters are given for both components (comp) by their mean (mu) and standard deviation (sigma), and scaled by factors lambda so as to result in an area of 1.0. Goodness of fit is given by the final log-likelihood value (loglik) of the optimisation algorithm, by Akaike's information criterion (AIC weighs loglik against the number of parameter estimates) and by the χ^2-value (a distance metric quantifying the difference between non-zero values of the histogram in Suppl. Fig. S3-A1 and the GMM fit). For both AIC and χ^2, lower values are better. AIC takes into account the model complexity, favouring less parameter estimates. χ^2 depends on the number of bins in the histogram (df is always the same for bimodal and unimodal GMMs per histogram), but not on model complexity.*

Shading indicates which model reaches the better fit (lower AIC value). Red shading indicates bimodal fits that essentially result in a unimodal distribution, owing to very low scaling parameter values (lambda) of the second Gaussian.

S3 B. Path parameters for experiment 2

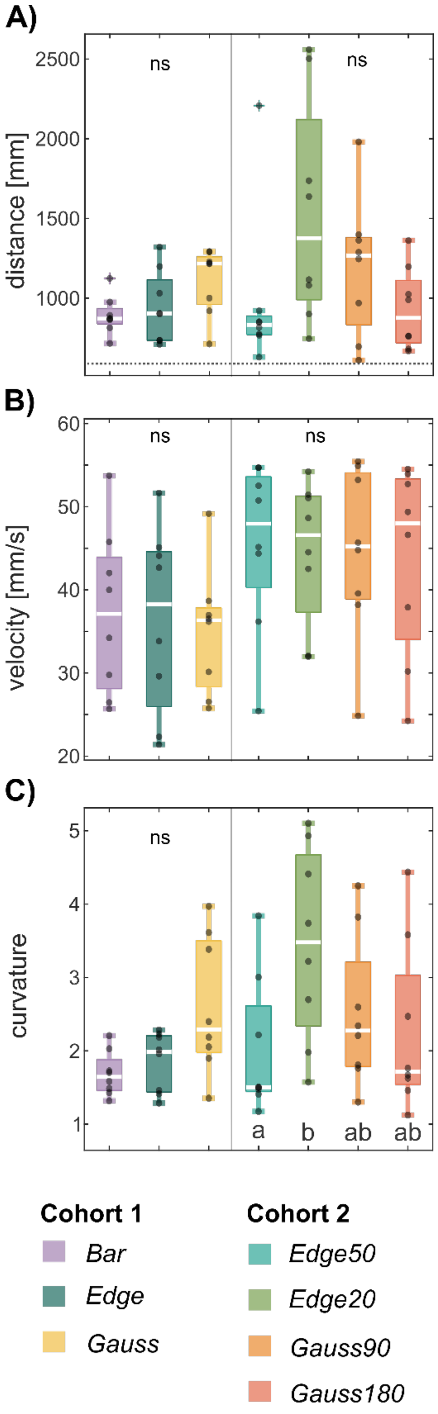

Suppl. Fig. S3-2 summarises three parameters of the animals' walking paths during experiment 2. The data are separated according to animal cohort. Cohort 1 comprised 8 animals that were tested on three visual patterns, a 90° Bar (with two contrast edges), a linear luminance gradient (Edge) with a single contrast edge, and a Gaussian luminance pattern of width 90° and σ = 10° (Gauss90) without contrast edge. Cohort 2 comprised further 8 animals that were tested on four visual patterns: two combinations of linear luminance gradients with contrast edges of equal Michelson contrast on either side (Edge20 with C_M = 20 %, and Edge50 with C_M = 50 %), and two Gaussian luminance patterns of width 90° and σ = 10° (Gauss90) or width 180° and σ = 30°.

Supplementary Figure S3-2: Visual patterns have no influence on walked distance, velocity or path curvature. *Experiments were done in two sets of eight animals. Statistical analysis was done separately for both cohorts. (**A**) Boxplots show the median walked distance per animal for three patterns in cohort 1 and four patterns in cohort 2. Dashed line indicates radius of the arena (600 mm) which is the minimum distance an animal had to walk to reach the arena wall. Friedman's test for matched samples showed no significant differences for either cohort. (**B**) Median translational velocity per animal. As in (A) medians were not significantly different for either cohort. (**C**) Median curvature per animal. Friedman's test revealed weakly significant differences for patterns of cohort 2 (p = 0.0158) but not for cohort 1. Bonferroni-corrected Wilcoxon's signed rank post-hoc tests revealed significantly increased path curvature for pattern Edge20 with low-contrast edges (p = 0.0078).*

Supplementary Material to Meschenmoser and Dürr (2025): *Contrast and luminance dependence of target choice and visual orientation in walking stick insects.*

S4: Supplement to discussion Figure 6.

Fig. 6 shows that the overall walking behaviour of stick insects differed when being presented with a 90° bar pattern with two contrast edges, as opposed to a 90° Gaussian pattern without contrast edges. Table S5 reports the total trial numbers of the 8 animals tested, along with the number of trials that terminated *at the wall*, n_W, and the number of trials that terminated *on target*, n_T. In case of the Gaussian pattern, 27 percent points less trials ended up at the wall, compared to the Bar pattern. At the same time, the fraction of animals that began to climb after reaching the wall was very similar for both patterns, irrespective of whether they were on target or not (bottom two rows in Table S5).

Supplementary Table S4*: Trial numbers and fraction of trials reported in Fig. 6.*

**Trials**		**Bar 90°**	**Gauss 90°**
**All trials**	Total	70; *69**	86; *84**
	At wall, n_W	77 % (54/70)	50 % (43/86)
	On target, n_T	69 % (48/70)	43 % (37/86)
**Climbs only**	Climbs, total	70 % (48/69*)	44 % (37/84*)
	Climbs, at wall	**89 % (48/54)**	**86 % (37/43)**
	Climbs, off target but at wall	**67 % (4/6)**	**60 % (3/5)**

** Climbs could not be assessed in one Bar- and two Gauss-trials. Numbers in parentheses give trial numbers used to calculate the percentages on the left.*

References

1 Jander, R .& Volk-Heinrichs, I. Das Strauch-spezifische Perceptor-System der Stabheuschrecke (*Carausius morosus*). *Z. vergl. Physiol.* **70**, 425–447 (1970).

2. Meyer-Rochow, V. B. & Keskinen, E. Post-embryonic photoreceptor development and dark/light adaptation in the stick insect *Carausius morosus* (Phasmida, Phasmatidae). *Applied Entomology and Zoology* **38**, 281–291 (2003).
